# *TMPRSS2* polymorphism (*rs12329760)* and the severity of the COVID-19 in Iranian population

**DOI:** 10.1371/journal.pone.0281750

**Published:** 2023-02-16

**Authors:** Arash Yaghoobi, Javad Safdari Lord, Javad Soltani Rezaiezadeh, Mir Saeed Yekaninejad, Masoumeh Amini, Pantea Izadi

**Affiliations:** 1 School of Medicine, Tehran University of Medical Sciences, Tehran, Iran; 2 Department of Medical Genetics, School of Medicine, Tehran University of Medical Sciences, Tehran, Iran; 3 Department of Epidemiology and Biostatistics, School of Public Health, Tehran University of Medical Sciences, Tehran, Iran; Imam Abdulrahman Bin Faisal University, SAUDI ARABIA

## Abstract

Severe Acute Respiratory Syndrome Coronavirus 2 (SARS-CoV-2) has been responsible for the recent pandemic since early 2020. Due to the wide range of clinical symptoms of this disease, from asymptomatic to severe and critical forms, it seems that genetic differences among patients, along with other factors (such as gender, age, and underlying diseases), can explain part of the variation in disease symptoms. The TMPRSS2 enzyme plays a vital role in the early stages of the interaction of the SARS-CoV-2 with the host cells by facilitating viral entry. There is a polymorphism in the *TMPRSS2* gene, called rs12329760(C to T) as a missense variant, which causes the replacement of valine to methionine in the TMPRSS2 protein at position 160. The present study investigated the association between the *TMPRSS2* genotype and the severity of the Coronavirus disease 2019 (COVID-19) in Iranian patients. The *TMPRSS2* genotype of 251 COVID-19 patients (151 patients with asymptomatic to mild and 100 patients with severe to critical symptoms) was detected on genomic DNA extracted from patients’ peripheral blood via the ARMS-PCR method. Our results showed a significant association between the minor T allele and the severity of the COVID-19 (*P-value* = 0.043) under the dominant and additive inheritance model. In conclusion, the results of this study showed that the T allele of the rs12329760 in the *TMPRSS2* gene is a risk allele for severe form of COVID-19 in Iranian patients in contrast to most previous studies on this variant in European ancestry populations which suggested this variant as a protective allele. Our results reiterate to the ethnic-specific risk alleles and hidden unknown complexity behind the host genetic susceptibility. However, further studies are needed to address the complex mechanisms behind the interaction of the TMPRSS2 protein and the SARS-CoV-2 and the role of rs12329760 polymorphism in determining the disease severity.

## Introduction

The Coronavirus Disease 2019 (COVID-19) pandemic was a major global crisis. On March 11, 2020, the World Health Organization declared the infection caused by Severe Acute Respiratory Syndrome Coronavirus 2 (SARS-CoV-2) a pandemic, becoming one of the deadliest pandemics of the last century [1]. By January2023, over 670 million infections and more than 6.5 million deaths have been recorded worldwide caused by the SARS-CoV-2 [2].

As the primary investigations showed, some background factors such as age (≥ 65 years), male gender, and underlying diseases affect the severity of COVID-19 presentation [3]. The mortality rate was higher among patients with obesity, type 2 diabetes, hypertension, cancer, and chronic kidney disease than in other groups [4, 5]. Although the SARS-CoV-2 enters the body via respiratory tracts, it spreads via circulation and can affect various systems (such as nervous, cardiovascular, reproductive, and gastrointestinal systems), leaving long-term complications in most of them [6]. In addition to the mentioned risk factors, host genetic factors may affect the susceptibility to COVID-19 and the disease’s severity [7, 8]. A higher concordance rate of COVID-19 in monozygotic twins than in dizygotic twins further supports the impact of genetic background on the clinical manifestations of COVID-19 [9]. Moreover, concerning the genetic background of the host, a highly complex network of different genes can affect the severity of SARS-CoV-2 infection, similar to other infectious diseases; most of them have little effect on the final phenotype; however, some specific genes and loci can have significant impacts on a patient’s phenotype in terms of infectious disease severity [10].

Several genome-wide association studies (GWAS) have been performed to unravel the association between the host genetic factors with the severity of COVID-19 [11]. Most of these associated loci are involved in the molecular processes such as viral entry into the host cells, immune system and inflammatory responses [12]. The *TMPRSS2* gene is one of the critical human genes in SARS-CoV-2 infection [13]. The SARS-CoV-2 interacts with some cellular proteins to enter the cell and make a successful infection: the spike glycoprotein on the surface of the virus is first cleaved by a set of cellular proteases, including the TMPRSS2 enzyme, and is converted into S1 and S2 subunits. The S1 part then binds to a receptor on the cell surface called ACE2. The S2 part is attached to the cell membrane and can enter the cell. The TMPRSS2 enzyme also facilitates viral entry into the cell by cleaving a part of the ACE2 receptor protein [14]. During the ACE2 processing, this enzyme competes with a metalloprotease (ADAM17), but the only effective molecular procedure for viral entry is the serine protease activity of TMPRSS2 [14]. The *TMPRSS2* encoding gene is located on chromosome 21 (21q22.3) and contains 15 exons and a reading frame for 492 amino acids [15]. This enzyme contains three main regions: its N-terminal region is located inside the cytoplasm, and next to it, there is an LDL receptor region that binds to calcium, and finally, there is a third region that is rich in the amino acid Cysteine (SRCR, which binds to other extracellular molecules). Its extracellular region is a catalytic region, activated by the autocatalytic process, entering the extracellular space and separating from the rest of the enzyme [15]. This enzyme has two isoforms, due to the alternative mRNA splicing process. Isoform 2 contains 492 amino acids, and isoform 1 contains 37 additional amino acid residues in the N-terminal region of this protein. The expression of this gene in the lungs, prostate, gastrointestinal tract, liver, heart, and cornea is higher than in the other tissues. Also, TMPRSS2 mRNA is detectable in the gallbladder and testis [16]. In the lung, it is mainly expressed in bronchial epithelial cells and to a lesser extent in the alveolar type 2 cells and alveolar macrophages [15]. The expression of this gene also increases with age in the lungs [17]. Also, the expression of the *TMPRSS2* gene increases in response to androgens [15]. No specific disease was developed in *TMPRSS2* knocked out mice, and no specific pathology was reported in humans related to the mutations in this gene [15]. This finding is likely due to the compensatory role of the other proteases in the Tmprss2-deficient mice. However, this enzyme plays different roles in several physiological and pathological processes (such as fertility, invasion of tumor cells, and blood clotting) [15–17].

It has been shown that some variants of the *TMPRSS2* gene can affect the severity of viral diseases such as influenza and COVID-19 [18]. Computational analysis has shown that among 21 SNPs in the *TMPRSS2* gene, the rs12329760 variant can affect the structure of this enzyme by forming a pocket in the protein structure [18]. The rs12329760 variant is a missense mutation (C to T) at position 589 of the *TMPRSS2* gene, resulting in the substitution of the methionine instead of the valine at position 197 of the protein (exon 7) in isoform 2and position 160 (exon 6) in isoform 1 of this enzyme [19]. This variant’s minor allele frequency (MAF) varies among different populations. For example, the minimum allele frequency for this variant has been seen in the Latin American population (15.33%). The maximum allele frequency has been found in the East Asian populations (38.38%) [20].

Since the results were not consistent among the studies performed on the association of the rs12329760 variant in the *TMPRSS2* gene with the severity of COVID-19 [21–25], this study aimed to investigate the association between rs12329760 polymorphism of the *TMPRSS2* gene with the severity of COVID-19 in the Iranian patients.

## Material and methods

### Study population

Two hundred and fifty-one COVID-19 patients referred to Farmanfarmayan Health center (Tehran, Iran) from September to December 2020 participated voluntarily in this study. The necessary condition for the inclusion of patients in this study was a positive COVID-19 PCR test of their nasopharyngeal swap samples. All participants were adults and signed written informed consent, then 5 ml peripheral blood samples were taken from patients in EDTA tubes as an anticoagulant. This study has been approved by the ethics committee of the school of medicine, Tehran University of Medical Sciences (Ethics code: IR.TUMS.MEDICINE.REC.1399.1091).

The control group comprised 151 patients with asymptomatic or mild forms of COVID-19. The case group included 100 patients with severe to critical symptoms (such as oxygen saturation level (SPO2) less than 90%, lung infiltration more than 50%, respiratory rate of more than 30 beats per minute, or organ failure) who needed hospitalization. All patients with known risk factors for severe COVID-19 (such as age over 65 years, diabetes, hypertension, heart failure, stroke, cancer chemotherapy, or immunodeficiency) were excluded from the case group.

### DNA extraction

Whole blood samples were used for genomic DNA extraction by the standard salting-out method described previously [26]. The quality and quantity of the purified DNA samples were evaluated by a Nano-Drop 2000TM spectrophotometer (Thermo Fisher Scientific, USA). The purified genomic DNA samples were stored at -20°C until genotyping by PCR.

### *TMPRSS2* genotyping

**Fig 1:**
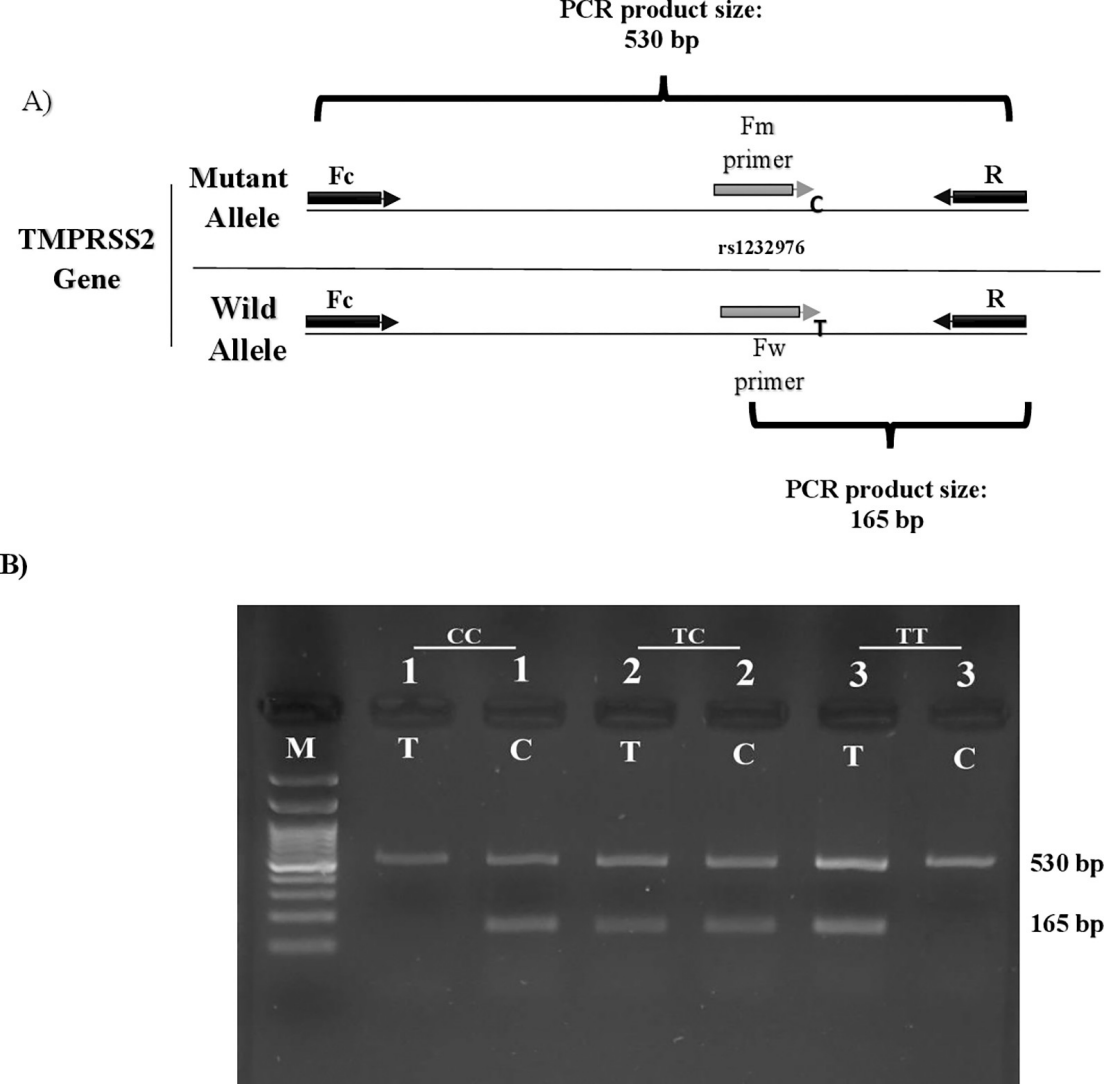
*TMPRSS2* rs12329760 genotyping by ARMS- PCR. A) Schematic diagram for primers positions in TMPRSS2 gene. In each PCR reaction, Fc and R primers produced 530 bp PCR product as internal control. The amplicon size for the allele-specific primers (Fw or Fm), was 165 bp. B) Amplified products of 3 patients after agarose gel electrophoresis shows three different genotypes. For each DNA sample, two ARMS-PCR reactions were conducted for detecting C and T alleles and the genotype of each patient defined above each pair of lanes. Lane M 100 bp ladder.

To detect rs12329760 variant alleles in the *TMPRSS2* gene, we analyzed all DNA samples with the amplification refractory mutation system PCR (ARMS-PCR) method. In this method, two allele-specific primers were used, one for the mutant allele (Fm) and the other for the wild-type allele (Fw) and as well as two internal control primers (R, Fc) to amplify the target DNA region. We conducted two separate ARMS-PCR reactions for each sample to detect C and T alleles. Primer sequences wereshown in Table 1. A total volume of 20 μl of the reaction mixture was prepared for each PCR reaction as follows: 12.5 μl of TEMPase Hot Start 2x Master Mix A BLUE (Amplicon, Denmark), 1.0 μl genomic DNA sample, 1.0 μl (5 pmol/ μl) of each control primers (R, Fc), 1 μl (5 pmol/ μl) of each allele-specific primer and 8.5 μl distilled water. No template control was performed for each experiment by adding 0.8 μl of water instead of genomic DNA. The amplification reaction was performed in ABI Veriti thermal cycler machine (Thermo Fisher Scientific, USA). The PCR program was carried out with an enzyme activation step at 95°C for 5 min, followed by 30 cycles (each cycle included: 30 s at 95°C, 30 s at 56°C, and 30 s at 72°C) and a final extension at 72°C for 7 min. The amplification products were separated by electrophoresis on 2% agarose gels (SinaClon BioScience, Iran) containing 0.5 mg/L DNA gel stain (SinaClon BioScience, Iran), and the image of amplified products was visualized under UV light and captured by gel documentation system (Syngene, UK). The amplicon size for internal control primers (FC, R primers) was 530 bp, and the allele-specific amplicon size was 165 bp (Fig 1).

**Table 1:** *ARMS PCR primers for TMPRSS2 rs12329760 genotyping*.

Primer name	Sequences
Fw (for wild-type allele)	5′- GGACCAAACTTCATCCTTCAAG-3′
Fm (for mutant allele)	5-′ GGACCAAACTTCATCCTTCAAA-3′
Fc (internal control)	5-′ ACGATACAAGTTGGGACAAGG-3′
R(common)	5-′ GTTTCTGCTGTCTGTTACTGTC-3′

### Statistical analysis

We used SPSS software (version 25) for performing *statistical analysis*. Chi-squared test (χ2) (Fisher’s exact test) was used to significantly compare the frequency of alleles and genotypes between case and control groups. Also, the association between *TMPRSS2* polymorphism and COVID-19 severity was analyzed in four different inheritance models using logistic regression, assuming gender and age as covariates using SNPstats online web tool software (https://www.snpstats.net/start.htm). Using logistic regression, we analyzed the interaction between genotypes and gender as a non-genetic factor on disease severity by adding an interaction term in SNPstats online web tool under four different inheritance models. The genotype frequencies in the study population were evaluated for concordance with Hardy Weinberg equilibrium using SNP Analyzer 2.0 software. The adjusted *P-value*, adjusted odds ratio, Akaike information criterion (AIC), and Bayesian information criterion (BIC) were calculated for each inheritance model with a 95% confidence interval to describe the strength of association using logistic regression by including age and gender as covariates. All *P-values* were considered statistically significant for *P*<0.05.

## Results

One hundred and fifty-one COVID-19 patients with asymptomatic or mild symptoms participated in the control group, and one hundred COVID-19 patients with severe or critical symptoms in the case group with a mean age of 38.39 (± 11.844) and 46.25 (± 10.07), respectively. In the control group, 61% of the patients were male. In the case group, 55% of the participants were males.

The *TMPRSS2* gene genotype was determined for 251 patient samples using the PCR method. Based on the presence of C and T alleles in the studied polymorphism, CC, CT, and TT genotypes are assumed. Then the frequency of each genotype and allele (Table 2) in the control and case groups was determined.

**Table 2:** Genotypes and alleles frequency of both control and case groups. P-values, odds ratio (OR) and 95% confidence intervals (95% CI).

		Control group n = 151 (%)	Case group n = 100 (%)	All COVID-19 n = 251(%)	*P-value*	OR (95% CI)
**Genotypes**	**CC**	103 (68.2%)	54 (54%)	157 (62.54)	0.074	-
	**CT**	43 (28.5%)	42(42%)	85 (33.86)		
	**TT**	5 (3.3%)	4 (4%)	9 (3.58)		
**Alleles**		**Control group n = 151 (%)**	**Case group n = 100 (%)**	**All COVID-19 n = 251(%)**	***P-value***	**OR (95% CI)**
	**C**	249 (82.5)	150 (75)	399 (79.5)	0.043	1.566 (1.012–2.423)
	**T**	53 (17.5)	50 (25)	103 (20.5)		

In the present study, the frequency of genotypes and alleles in the entire patient population is under Hardy-Weinberg equilibrium (*P-value* = 0.544). In the case and control groups, genotypes are also under Hardy Weinberg equilibrium (*P-value* = 0.230, *P-value* = 0.938, respectively).

**Fig 2:**
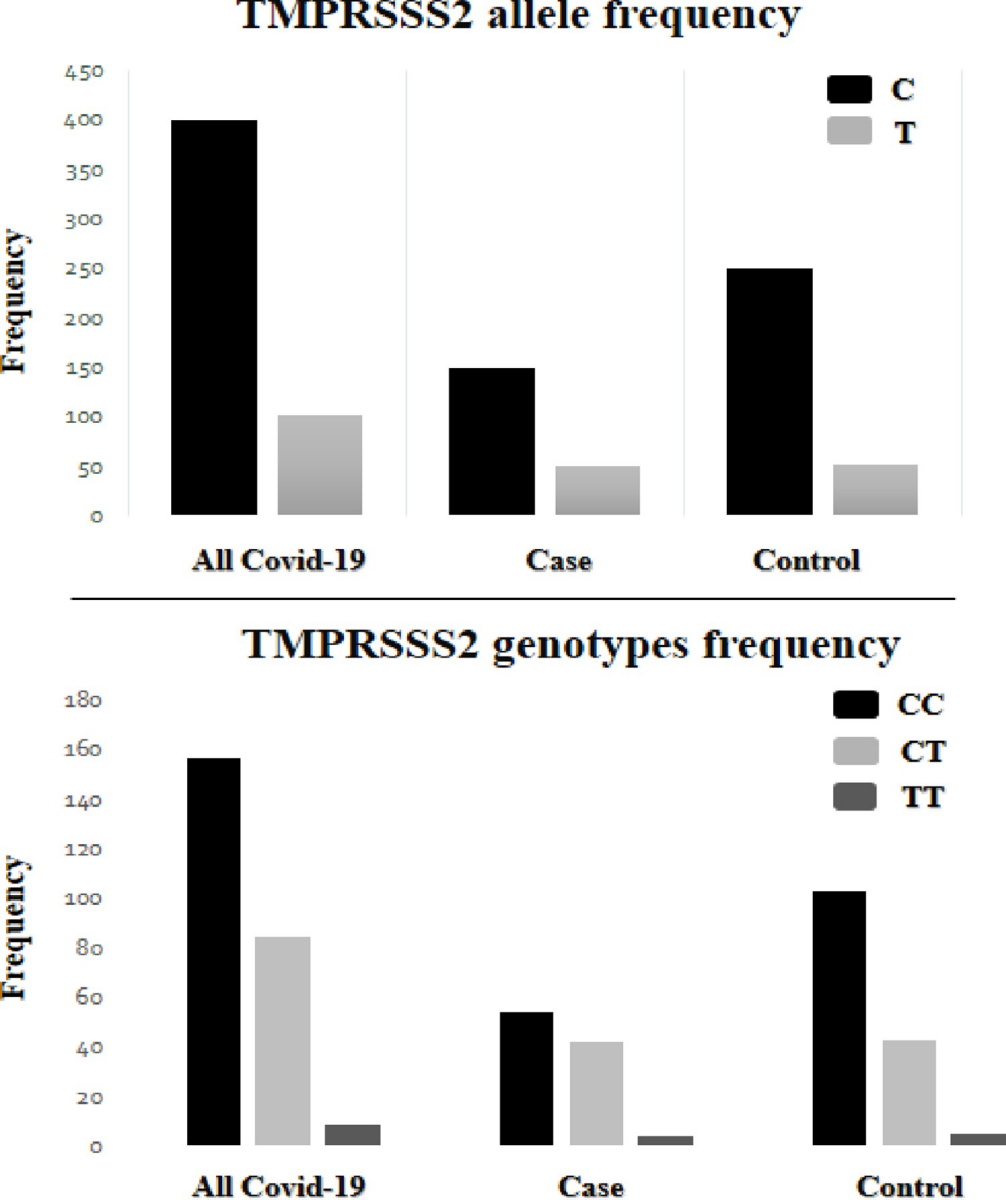
Allelic and genotypic frequency distributions of *TMPRSS2* polymorphism in case and control groups and all COVID-19 patients (All COVID-19 patients = case + control).

Statistical analysis between the association of the C and T alleles with the severity of symptoms showed a statistically significant difference (*P-value* = 0.043, OR = 1.566 (1.012–2.423). The frequency of the C allele decreased from 82.5% in the control group to 75% in the case group. The frequency of the minor T allele increased from 17.5% in the control group to 25% in the case group. Also, the total frequency of the T and C allele among 251 patients was 20.5% and 79.5%, respectively. Statistical analysis did not show a statistically significant difference between the distribution of three genotypes and the severity of the disease. The frequency of the CC genotype decreased from 68.2% in the control group to 54% in the case group. Also, the frequency of the CT genotype increased from 28.5% in the control group to 42% in the case group. Finally, the frequency of the TT genotype increased from 3.3% in the control group to 4% in the case group. The frequencies of genotypes and alleles are shown graphically in the case and control groups and all patients in Fig 2.

We performed logistic regression modeling by including gender and age as covariates to analyze the results under different inheritance models. Statistical analysis did not show a statistically significant difference between the distribution of three genotypes and the severity of the disease under the Co-dominant model (*P-value* = 0.056). A statistically significant difference was found in the dominant model by comparing the CC genotype and the sum of CT + TT genotypes (*P-value* = 0.016, OR = 1.97 (1.13–3.43)) in the case and control groups. In the recessive model, by comparing the TT genotype with the sum of CC + CT genotypes in the case and control groups, no statistically significant difference was found (*P-value* = 0.56, OR = 1. 52 (0.38–6.12)). Finally, in the log-additive model, a statistically significant difference was also found (*P-value* = 0.024, OR = 1.73(1.07–2.79). The results of these analyzes are shown in Table 3. Akaike information criterion (AIC) and Bayesian information criterion (BIC) were calculated to analyze the fitness of models. The fittest model for explaining our results was the dominant inheritance model.

**Table 3:** Analysis of *TMPRSS2* genotypes association with COVID-19 disease severity under five different inheritance models adjusted by gender and age. Adjusted P-values, adjusted odds ratio (OR), 95% confidence intervals (95% CI), AIC and BIC.

Inheritance model	Genotype	Control group N (%)	Case group N (%)	Adjusted OR (95% CI)	Adjusted *P-value*	AIC	BIC
**Co-dominant**	CC	103 (68.2%)	54 (54%)	1.00	0.056	313.4	331
	CT	43 (28.5%)	42(42%)	1.97 (1.11–3.49)			
	TT	5 (3.3%)	4 (4%)	1.96 (0.48–8.04)			
**Dominant**	CC	103 (68.2%)	54 (54%)	1.00	**0.016**	**311.4**	**325.5**
	CT+TT	48 (31.8%)	46 (46%)	**1.97 (1.13–3.43)**			
**Recessive**	CC+CT	146 (96.7%)	96 (96%)	1.00	0.56	316.8	330.9
	TT	5 (3.3%)	4 (4%)	1.52 (0.38–6.12)			
**Log-additive**	-	-	-	1.73 (1.07–2.79)	0.024	312	326.1

N: number, OR: Odds ratio, CI: Confidence interval, AIC: Akaike information criterion, BIC: Bayesian information criterion

Statistical analyses of genotypes’ interaction with gender as a non-genetic covariate and their association with disease severity were performed for four different inheritance models using logistic regression, and their interaction *P-values* were calculated. Also, odds ratio and *P-values* were calculated for these four models separately by classifying patients first by the SNP and then by sex using the Trend test for the interaction. None of the models showed statistically significant interactions between gender and genotypes regarding disease severity. Also, the Trend test for the interaction showed no statistically significant interaction between genotypes and gender, neither for gender within genotype analyses nor genotype within gender analyses in all four models (Table 4).

**Table 4:** Test statistics for interaction between genotypes and gender in determining the COVID-19 severity.

Inheritance model	Interaction *P-value*	Trend test *P-value* (Gender within genotype)	Trend test *P-value* (genotype within gender)
**Co-dominant**	0.76	0.76	0.76
**Dominant**	0.97	0.97	0.97
**Recessive**	0.49	0.49	0.49
**Log-additive**	0.76	0.76	0.76

Statistical analysis did not show a significant relationship between patients’ sex and the severity of COVID-19 symptoms (*P-value* = 0.252).

## Discussion

Following the outbreak of COVID-19, observations showed differences in the severity of symptoms. Host genetic background along with other risk factors (such as gender, age and comorbidities) can explain part of the observed variation in COVID-19 symptoms. The rs12329760 variant of the *TMPRSS2* gene is located in the coding part of the gene. According to the predictions of some bioinformatics software, this variant can decrease enzyme activity and stability; hence activation of the SARS-CoV-2 and its entry into host cells are diminished [18, 27]. Therefore, this variant has been predicted to have a protective effect against SARS-CoV-2 infection. Interestingly, this variant has been proposed as one of the candidate gene variants in justifying the high incidence and COVID-19-related mortality rate in the Italian population compared to the other European and Asian countries [28].

Considering the role of this variant in the entry of the virus into the host cell, we aimed to investigate the association between the *TMPRSS2* rs12329760 variant and the severity of COVID-19 in the Iranian population. Two recent studies on Iranian patients have uncovered the roles of some polymorphisms in different genes in determining the COVID-19 severity [29, 30]. Our study showed that the T allele of this variant had a statistically significant association with the severity of COVID-19 symptoms (*P-value* = 0.043, OR = 1.566, 95%CI = 1.012–2.423). Under the fittest (dominant) model, the risk of developing a severe form of COVID-19 almost increased by approximately 2-folds (OR = 1.97, 95%CI = 1.13–3.43).

Interestingly, the result of our investigation in Iranian patients is in contrast to most previous studies. Some studies have consistently concluded that the minor T allele of this variant is associated with a reduction in the severity of symptoms and incidence of the COVID-19 [22, 23, 31, 32]. For instance, in a recent study, the authors showed the association of the T allele with the reduced likelihood of developing severe COVID-19 in the UK population [31]. In a recent systematic review, the T allele was associated with a reduction in the severity of the COVID-19 [33]. In another investigation in Italian patients, the authors suggested this variant as a protective factor against the COVID-19, especially among young men and older women [21].

However, in some studies, the authors have failed to find any association between this variant of the *TMPRSS2* gene and COVID-19 severity [24, 25, 34]. For example, in two case-control studies, the authors found no statistically significant association between the severity of the COVID-19 and the rs12329760 variant in Indonesian and German patients [24, 25]. Also, in another investigation in Chinese patients, there was no statistically significant association between the mentioned variant and disease severity [32]. Finally, in two recent studies with population ancestries that are more similar to our study population, results showed that the T allele of this variant is associated with an increased risk of SARS-CoV-2 infection and disease severity and their findings are in accordance with our results [35, 36]. The differences in ethnicity and existence of haplotype blocks with special combination of other variants in risk loci, can be suggested for inconsistency between the findings of investigations. It can be suggested that the T allele effect on the COVID-19 severity may depend on the patient’s ancestry. *TMPRSS2* gene has various SNPs and their combinatory effects may influence the disease outcome in a complex way, causing these conflicting results. More investigation are needed to clarify this issue.

Although, computational analysis about the influence of the rs12329760 variant on TMPRSS2 enzyme function [18] predicted that it could theoretically increase the viral load by reducing the viral entrance to the host cells, the actual link between viral load and the disease severity is still poorly understood [37]. For example, an investigation, showed an association between this variant and the viral load despite the lack of association between this variant and the disease severity [24]. Therefore, there may be no simple relationship between viral load and disease severity and the molecular mechanism behind the role of rs12329760 in disease severity must be clarified in future investigations.

Regulation of the *TMPRSS2* gene expression by androgen hormones has been suggested as one of the possible mechanisms explaining the difference in the severity of the COVID-19 between men and women. However, in one study, the overall expression of the *TMPRSS2* gene in men did not show a significant increase compared to women [38]. In our study, additional investigation in any four inheritance models did not show a statistically significant interaction between gender and genotypes regarding disease severity. This inconsistency in findings in different studies may be because, unlike other tissues in the body, the expression of the *TMPRSS2* gene in the lung does not appear to be regulated by androgen hormones [39].

Our study had several limitations: our sample size is relatively small. Also, we did not study the variant’s effect on TMPRSS2 protein function. However, we tried to increase the power of our association study by excluding main comorbidities and old age as confounding factors in the case group. Our findings may lead to a fundamental revision in the current understanding of the TMPRSS2 role in COVID-19 severity based on an ethnic-based approach in order to recognize high-risk population and may open up a new window for better understanding the interaction of the host cells with COVID-19, which, in turn, can lead to the development of new therapies for high-risk patients.

In conclusion, our findings showed a statistically significant association between the rs12329760 T allele as a risk allele and the severity of the COVID-19 in contrast to some previous investigations. Our finding adds new insight to the literature on the role of *TMPRSS2* in COVID-19 severity and point to the ethnic-specific risk alleles and hidden unknown complexity behind the host genetic susceptibility to SARS-CoV-2 infection. More sample sizes should be used in subsequent studies to confirm our findings. Because our study consisted of only Iranian patients, future multinational studies should be conducted to clarify the influence of ethnicity on this polymorphism’s effect on the disease severity. Also, the effect of this variant on *TMPRSS2* gene expression and protein activity should be addressed in future functional studies.

## Supporting information

S1 Data (XLSX)

S1 Raw images (PDF)

## References

[1] Azarpazhooh MR, Morovatdar N, Avan A, Phan TG, Divani AA, Yassi N, et al. COVID-19 Pandemic and Burden of Non-Communicable Diseases: An Ecological Study on Data of 185 Countries. J Stroke Cerebrovasc Dis. 2020;29(9):105089. doi: 10.1016/j.jstrokecerebrovasdis.2020.105089 32807484 PMC7315949

[2] COVID live—Coronavirus statistics—worldometer [Internet]. Worldometers.info. [cited 2022 Aug 3]. [Available from: https://www.worldometers.info/coronavirus/

[3] Drake TM, Riad AM, Fairfield CJ, Egan C, Knight SR, Pius R, et al. Characterisation of in-hospital complications associated with COVID-19 using the ISARIC WHO Clinical Characterisation Protocol UK: a prospective, multicentre cohort study. The Lancet. 2021;398(10296):223–37. 10.1016/S0140-6736(21)00799-6 PMC8285118 34274064

[4] Ng WH, Tipih T, Makoah NA, Vermeulen J-G, Goedhals D, Sempa JB, et al. Comorbidities in SARS-CoV-2 patients: a systematic review and meta-analysis. Mbio. 2021;12(1):e03647–20. doi: 10.1128/mBio.03647-20 33563817 PMC7885108

[5] Senapati S, Kumar S, Singh AK, Banerjee P, Bhagavatula S. Assessment of risk conferred by coding and regulatory variations of TMPRSS2 and CD26 in susceptibility to SARS-CoV-2 infection in human. Journal of genetics. 2020;99(1):1–5. doi: 10.1007/s12041-020-01217-7 32661206 PMC7280172

[6] Rabaan AA, Smajlović S, Tombuloglu H, Ćordić S, Hajdarević A, Kudić N, et al. SARS‐CoV‐2 infection and multi-organ system damage: a review. Biomolecules and Biomedicine. 2023;23(1):37–52. doi: 10.17305/bjbms.2022.7762 36124445 PMC9901898

[7] Hou Y, Zhao J, Martin W, Kallianpur A, Chung MK, Jehi L, et al. New insights into genetic susceptibility of COVID-19: an ACE2 and TMPRSS2 polymorphism analysis. BMC medicine. 2020;18(1):1–8. 32664879 10.1186/s12916-020-01673-z PMC7360473

[8] Latini A, Agolini E, Novelli A, Borgiani P, Giannini R, Gravina P, et al. COVID-19 and Genetic Variants of Protein Involved in the SARS-CoV-2 Entry into the Host Cells. Genes. 2020;11(9):1010. doi: 10.3390/genes11091010 32867305 PMC7565048

[9] de Castro MV, Silva MVR, Naslavsky MS, Santos KS, Magawa JY, Neto EC, et al. [Pre-print].COVID-19 in twins: What can we learn from them? 2021:[2021.09.29.21263145 p.]. Available from: https://www.medrxiv.org/content/medrxiv/early/2021/10/02/2021.09.29.21263145.full.pdf.

[10] Van Sluijs L, Pijlman G, Kammenga J. Why do Individuals Differ in Viral Susceptibility? A Story Told by Model Organisms. Viruses. 2017;9(10):284. doi: 10.3390/v9100284 28973976 PMC5691635

[11] Karlsen TH. Understanding COVID-19 through genome-wide association studies. Nature Genetics. 2022;54(4):368–9. doi: 10.1038/s41588-021-00985-x 35410380

[12] Velavan TP, Pallerla SR, Rüter J, Augustin Y, Kremsner PG, Krishna S, et al. Host genetic factors determining COVID-19 susceptibility and severity. EBioMedicine. 2021;72:103629. doi: 10.1016/j.ebiom.2021.103629 34655949 PMC8512556

[13] Abdollahi S, Izadi P. TMPRSS2 As an Influential Human Gene for COVID-19. 2020;4(1):e119384.

[14] Heurich A, Hofmann-Winkler H, Gierer S, Liepold T, Jahn O, Pohlmann S. TMPRSS2 and ADAM17 Cleave ACE2 Differentially and Only Proteolysis by TMPRSS2 Augments Entry Driven by the Severe Acute Respiratory Syndrome Coronavirus Spike Protein. Journal of Virology. 2014;88(2):1293–307. doi: 10.1128/JVI.02202-13 24227843 PMC3911672

[15] Kim TS, Heinlein C, Hackman RC, et al. Phenotypic analysis of mice lacking the Tmprss2-encoded protease. Mol Cell Biol 2006;26:965–75. doi: 10.1128/MCB.26.3.965-975.2006 16428450 PMC1347042

[16] Dong M, Zhong J, Ma X, Tan J, Chen L, Liu S, et al. *ACE2, TMPRSS2* distribution and extrapulmonary organ injury in patients with COVID-19. Biomedicine & Pharmacotherapy. 2020; 131:110678. doi: 10.1016/j.biopha.2020.110678 32861070 PMC7444942

[17] Schuler BA, Habermann AC, Plosa EJ, Taylor CJ, Jetter C, Negretti NM, et al. Age-determined expression of priming protease TMPRSS2 and localization of SARS-CoV-2 in lung epithelium. The Journal of clinical investigation. 2021;131(1). doi: 10.1172/JCI140766 33180746 PMC7773394

[18] Paniri A, Hosseini MM, Akhavan-Niaki H. First comprehensive computational analysis of functional consequences of TMPRSS2 SNPs in susceptibility to SARS-CoV-2 among different populations. Journal of Biomolecular Structure and Dynamics. 2021;39(10):3576–93. doi: 10.1080/07391102.2020.1767690 32410502 PMC7284145

[19] Zarubin A, Stepanov V, Markov A, Kolesnikov N, Marusin A, Khitrinskaya I, et al. Structural variability, expression profile, and pharmacogenetic properties of TMPRSS2 gene as a potential target for COVID-19 therapy. Genes. 2021;12(1):19. 10.3390/genes12010019 PMC7823984 33375616

[20] Lee I-H, Lee J-W, Kong SW. A survey of genetic variants in SARS-CoV-2 interacting domains of ACE2, TMPRSS2 and TLR3/7/8 across populations. Infection, Genetics and Evolution. 2020;85:104507. doi: 10.1016/j.meegid.2020.104507 32858233 PMC7448771

[21] Monticelli M, Hay Mele B, Benetti E, Fallerini C, Baldassarri M, Furini S, et al. Protective Role of a TMPRSS2 Variant on Severe COVID-19 Outcome in Young Males and Elderly Women. Genes. 2021;12(4):596. doi: 10.3390/genes12040596 33921689 PMC8073081

[22] Ravikanth V, Sasikala M, Naveen V, Latha SS, Parsa KVL, Vijayasarathy K, et al. A variant in TMPRSS2 is associated with decreased disease severity in COVID-19. Meta gene. 2021;29:100930. doi: 10.1016/j.mgene.2021.100930 34075330 PMC8161869

[23] Andolfo I, Russo R, Lasorsa VA, Cantalupo S, Rosato BE, Bonfiglio F, et al. Common variants at 21q22.3 locus influence MX1 and TMPRSS2 gene expression and susceptibility to severe COVID-19. iScience. 2021;24(4):102322. doi: 10.1016/j.isci.2021.102322 33748697 PMC7968217

[24] Wulandari L, Hamidah B, Pakpahan C, Damayanti NS, Kurniati ND, Adiatmaja CO, et al. Initial study on TMPRSS2 p. Val160Met genetic variant in COVID-19 patients. Human genomics. 2021;15(1):1–9. 34001248 10.1186/s40246-021-00330-7 PMC8127183

[25] Schönfelder K, Breuckmann K, Elsner C, Dittmer U, Fistera D, Herbstreit F, et al. Transmembrane serine protease 2 Polymorphisms and Susceptibility to Severe Acute Respiratory Syndrome Coronavirus Type 2 Infection: A German Case-Control Study. Frontiers in Genetics. 2021;12:667231. doi: 10.3389/fgene.2021.667231 33968142 PMC8097083

[26] MWer S, Dykes D, Polesky H. A simple salting out procedure for extracting DNA from human nucleated cells. Nucleic acids res. 1988;16(3):1215. doi: 10.1093/nar/16.3.1215 3344216 PMC334765

[27] Vishnubhotla R, Vankadari N, Ketavarapu V, Amanchy R, Avanthi S, Bale G, et al. [Pre-print].Genetic variants in TMPRSS2 and Structure of SARS-CoV-2 spike glycoprotein and TMPRSS2 complex 2020:[2020.06.30.179663 p.]. Available from: https://www.biorxiv.org/content/biorxiv/early/2020/06/30/2020.06.30.179663.full.pdf.

[28] Strope JD, Pharmd CHC, Figg WD. TMPRSS2: Potential Biomarker for COVID‐19 Outcomes. The Journal of Clinical Pharmacology. 2020;60(7):801–7. doi: 10.1002/jcph.1641 32437018 PMC7280622

[29] Safdari Lord J, Soltani Rezaiezadeh J, Yekaninejad MS, Izadi P. The association of APOE genotype with COVID-19 disease severity. Scientific reports. 2022;12(1):1–6. 35931737 10.1038/s41598-022-17262-4 PMC9356041

[30] Soltani Rezaiezadeh J, Safdari Lord J, Yekaninejad MS, Izadi P. The association of ACE I/D polymorphism with the severity of COVID-19 in Iranian patients: A case-control study. Human Gene. 2022;34:201099. 10.1016/j.humgen.2022.201099 PMC9364667 37521445

[31] David A, Parkinson N, Peacock TP, Pairo-Castineira E, Khanna T, Cobat A, et al. A common TMPRSS2 variant has a protective effect against severe COVID-19. Current research in translational medicine. 2022;70(2):103333. doi: 10.1016/j.retram.2022.103333 35104687 PMC8743599

[32] Jeon S, Blazyte A, Yoon C, Ryu H, Jeon Y, Bhak Y, et al. Regional TMPRSS2 V197M allele frequencies are correlated with COVID-19 case fatality rate. Molecules and cells. 2021;44(9):680. 34588322 10.14348/molcells.2021.2249 PMC8490206

[33] Saengsiwaritt W, Jittikoon J, Chaikledkaew U, Udomsinprasert W. Genetic polymorphisms of ACE1, ACE2, and TMPRSS2 associated with COVID‐19 severity: A systematic review with meta‐analysis. Reviews in Medical Virology. 2022:e2323. doi: 10.1002/rmv.2323 34997794

[34] Wang F, Huang S, Gao R, Zhou Y, Lai C, Li Z, et al. Initial whole-genome sequencing and analysis of the host genetic contribution to COVID-19 severity and susceptibility. Cell discovery. 2020;6(1):1–16. doi: 10.1038/s41421-020-00231-4 33298875 PMC7653987

[35] Rokni M, Heidari Nia M, Sarhadi M, Mirinejad S, Sargazi S, Moudi M, et al. Association of TMPRSS2 Gene Polymorphisms with COVID-19 Severity and Mortality: a Case-Control Study with Computational Analyses. Applied biochemistry and biotechnology. 2022:1–20. 10.1007/s12010-022-03885-w PMC8986508 35386063

[36] Abdelsattar S, Kasemy Z, Ewida S, Abo-Elsoud R, Zytoon A, Abdelaal G, et al. ACE2 and TMPRSS2 SNPs as Determinants of Susceptibility to, and Severity of, a COVID-19 Infection. British Journal of Biomedical Science. 2022;79:10238. doi: 10.3389/bjbs.2021.10238 35996506 PMC8915702

[37] Dadras O, Afsahi AM, Pashaei Z, Mojdeganlou H, Karimi A, Habibi P, et al. The relationship between COVID‐19 viral load and disease severity: A systematic review. Immunity, inflammation and disease. 2022;10(3):e580. doi: 10.1002/iid3.580 34904379 PMC8926507

[38] Asselta R, Paraboschi EM, Mantovani A, Duga S. ACE2 and TMPRSS2 variants and expression as candidates to sex and country differences in COVID-19 severity in Italy. Aging. 2020;12(11):10087–98. doi: 10.18632/aging.103415 32501810 PMC7346072

[39] Li F, Han M, Dai P, Xu W, He J, Tao X, et al. Distinct mechanisms for TMPRSS2 expression explain organ-specific inhibition of SARS-CoV-2 infection by enzalutamide. Nature communications. 2021;12(1):1–14. 10.1038/s41467-021-21171-x PMC7870838 33558541

