# Supplementary material for: TMPRSS2 polymorphism (rs12329760) and the severity of the COVID-19 in Iranian population
Source: PLoS One. 2023 Feb 16;18(2):e0281750. doi: 10.1371/journal.pone.0281750 (PMC9934348; doi:10.1371/journal.pone.0281750)
Supplement: S1 Raw images — (PDF) [file pone.0281750.s002.pdf]

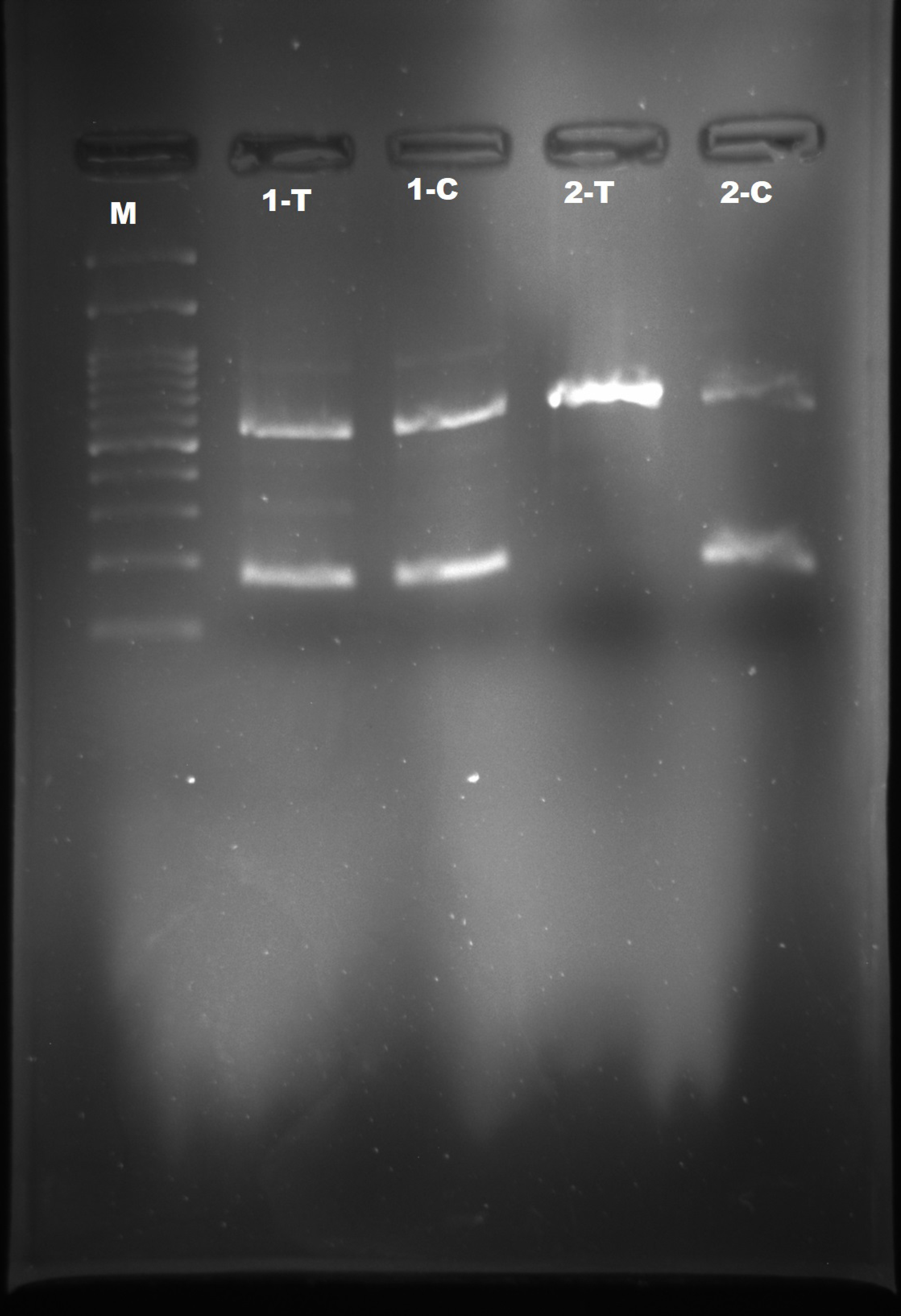
M
1-T
1-C
2-T
2-C

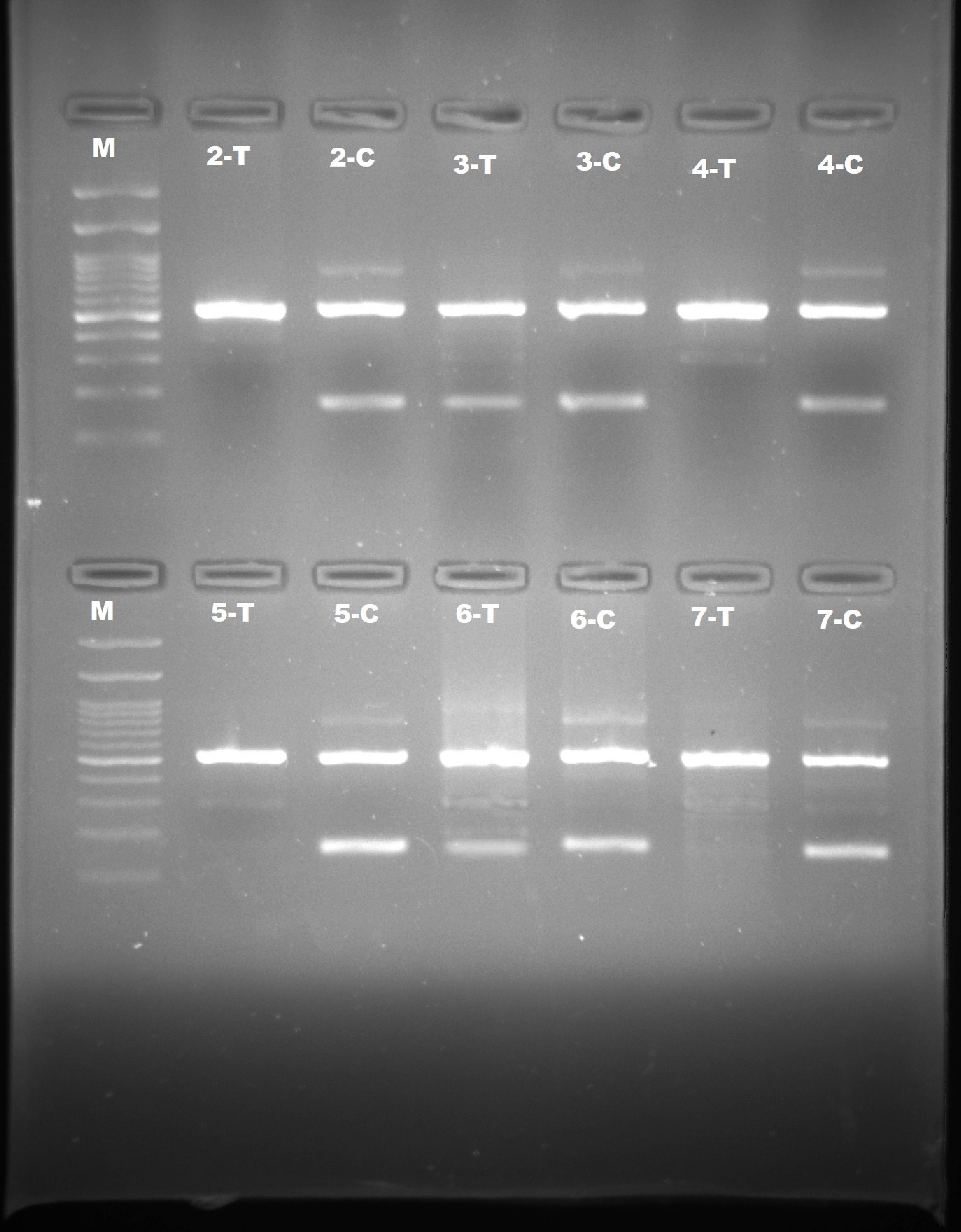
M
2-T
2-C
3-T
3-C
4-T
4-C
M
5-T
5-C
6-T
6-C
7-T
7-C

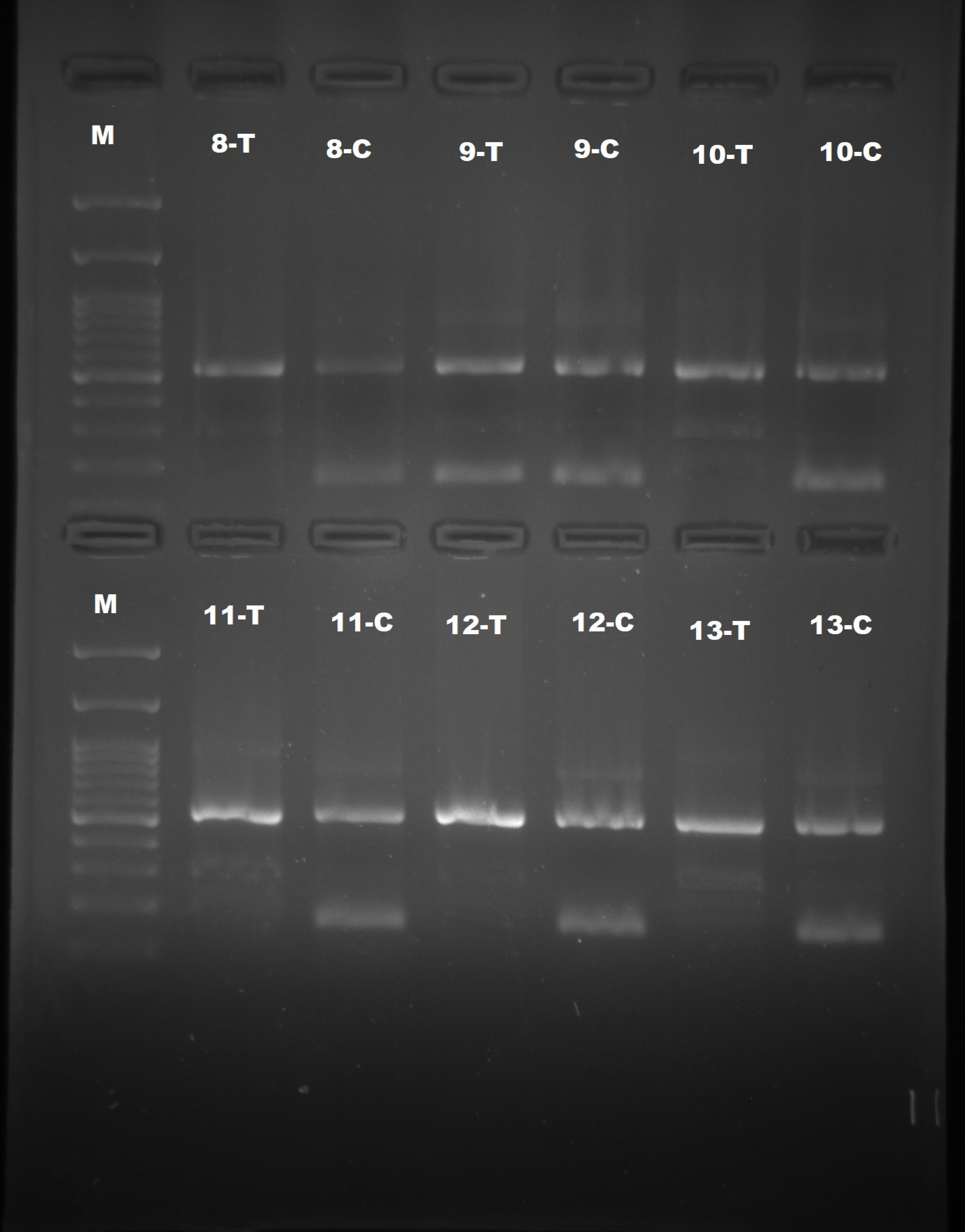
M
8-T
8-C
9-T
9-C
10-T
10-C
M
11-T
11-C
12-T
12-C
13-T
13-C

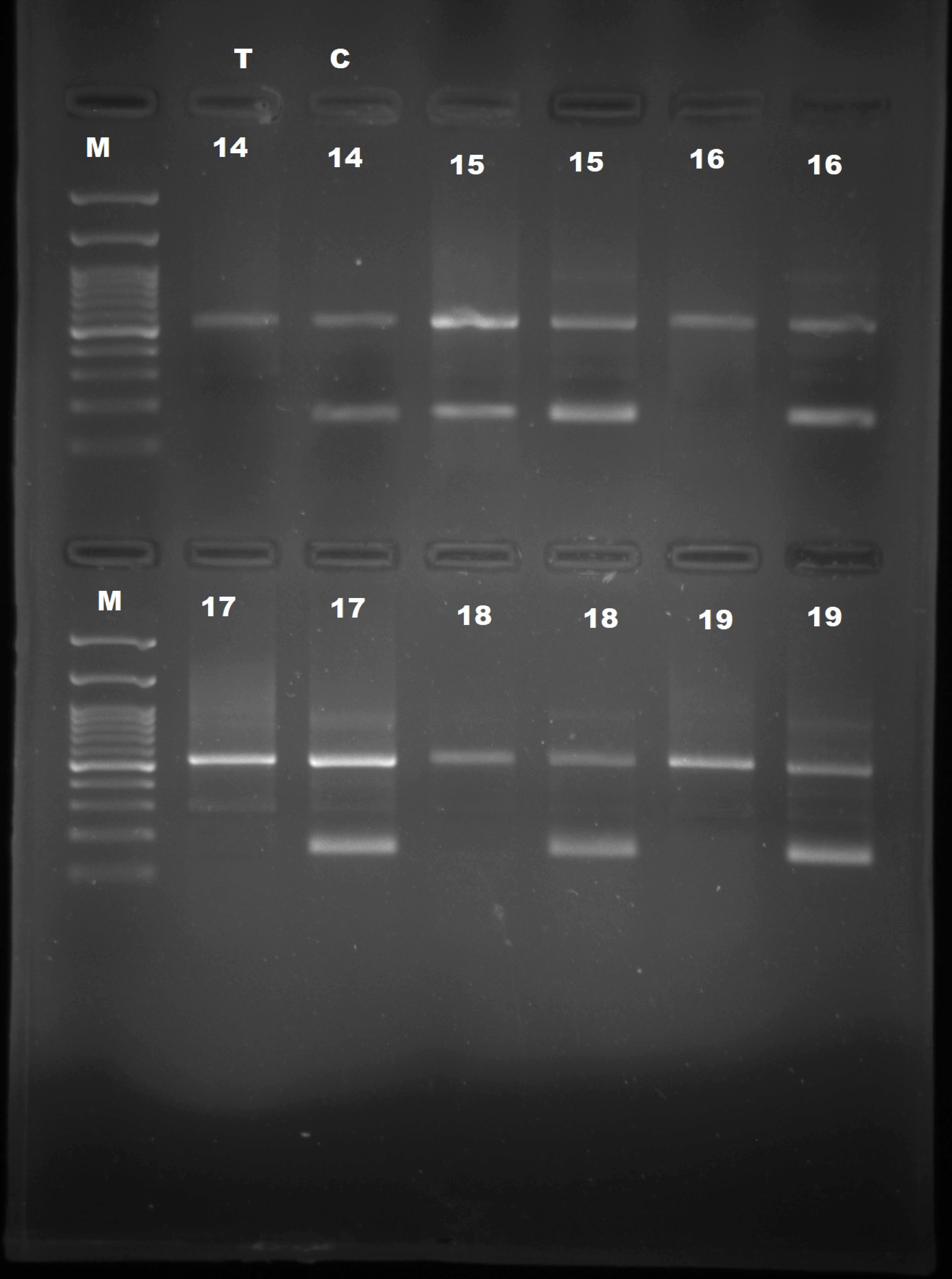

T
C
M
14
14
15
15
16
16
M
17
17
18
18
19
19

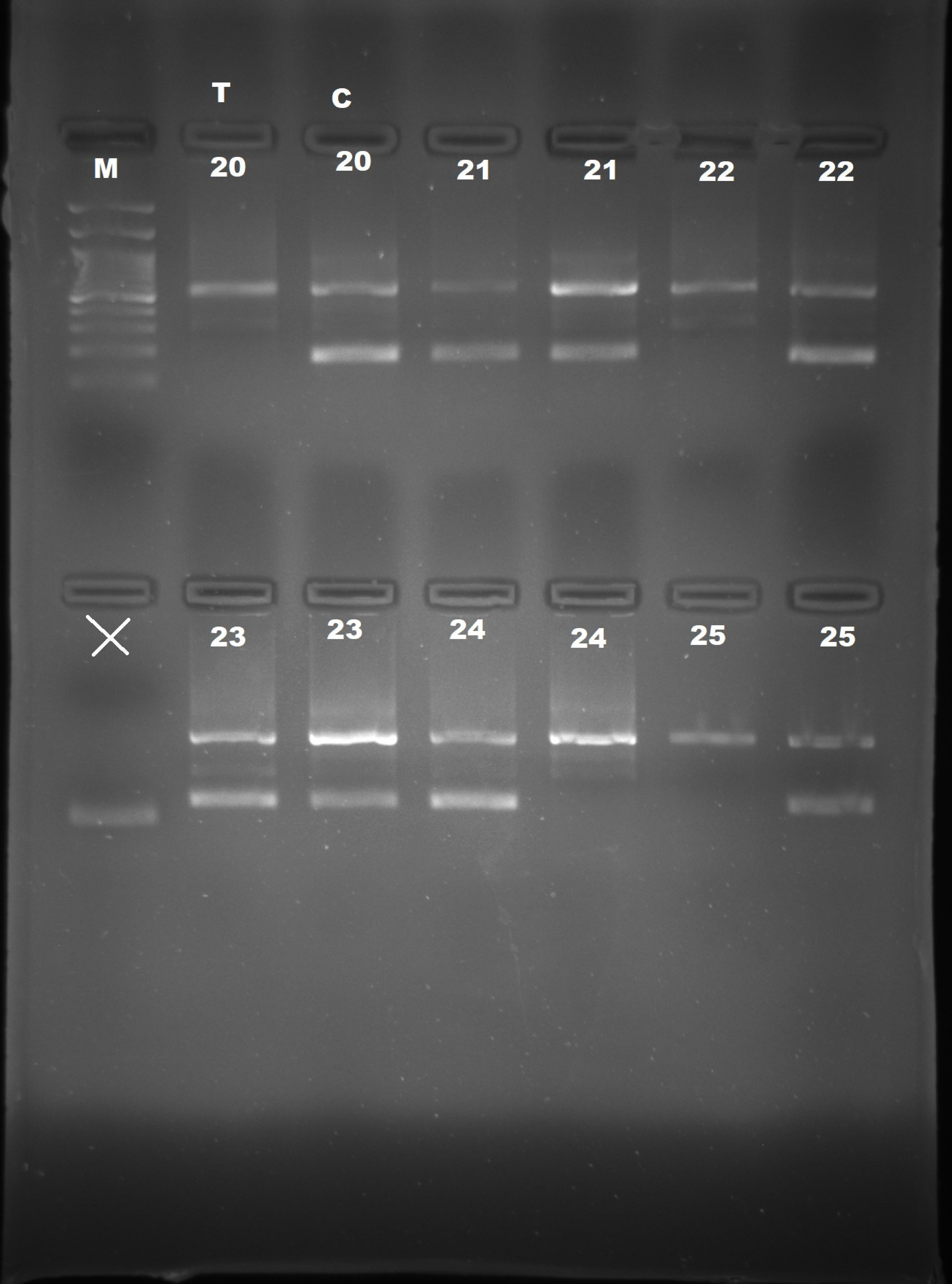
T
C
M
20
20
21
21
22
22
23
23
24
24
25
25

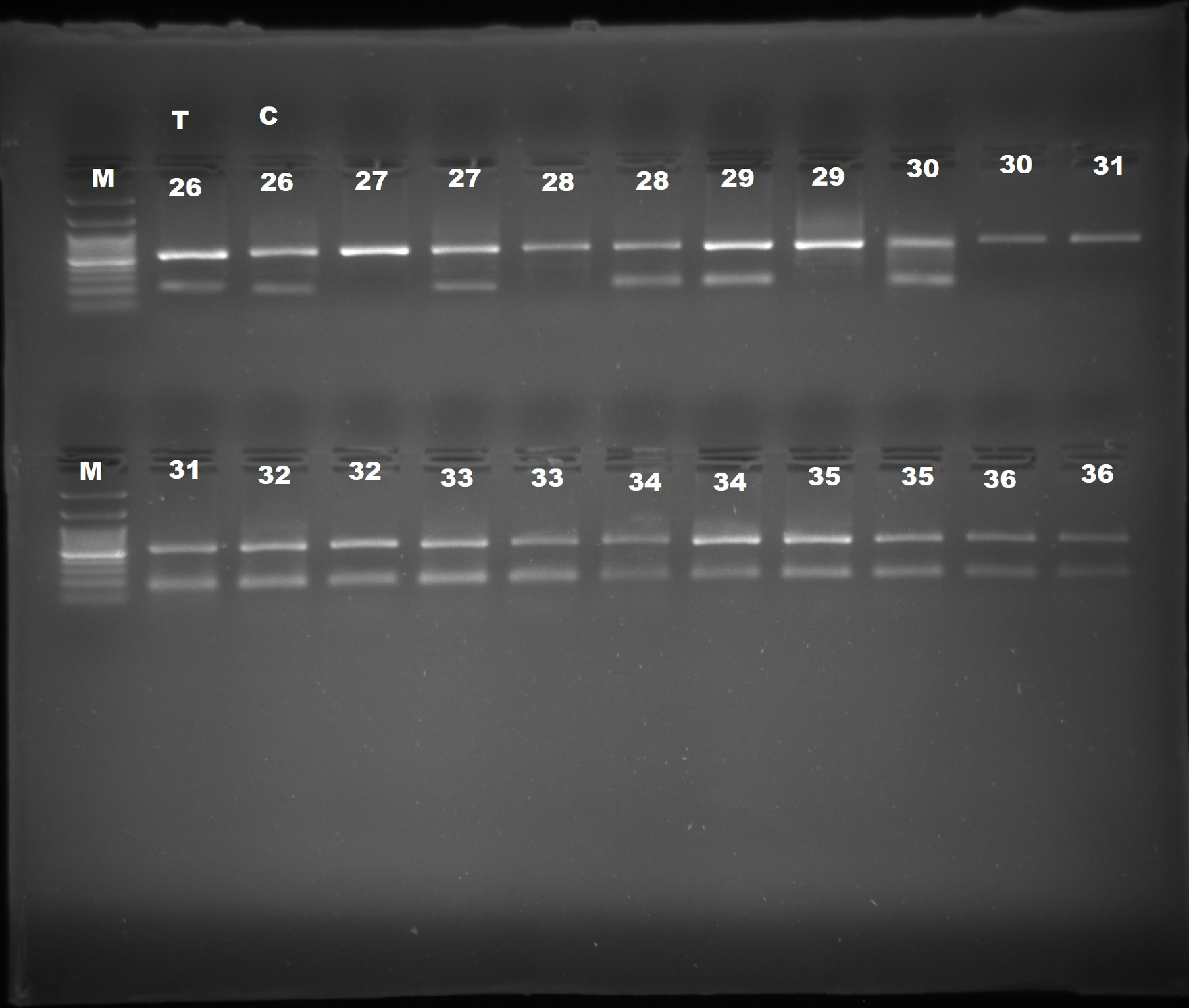
T
C
M
26
26
27
27
28
28
29
29
30
30
31
M
31
32
32
33
33
34
34
35
35
36
36

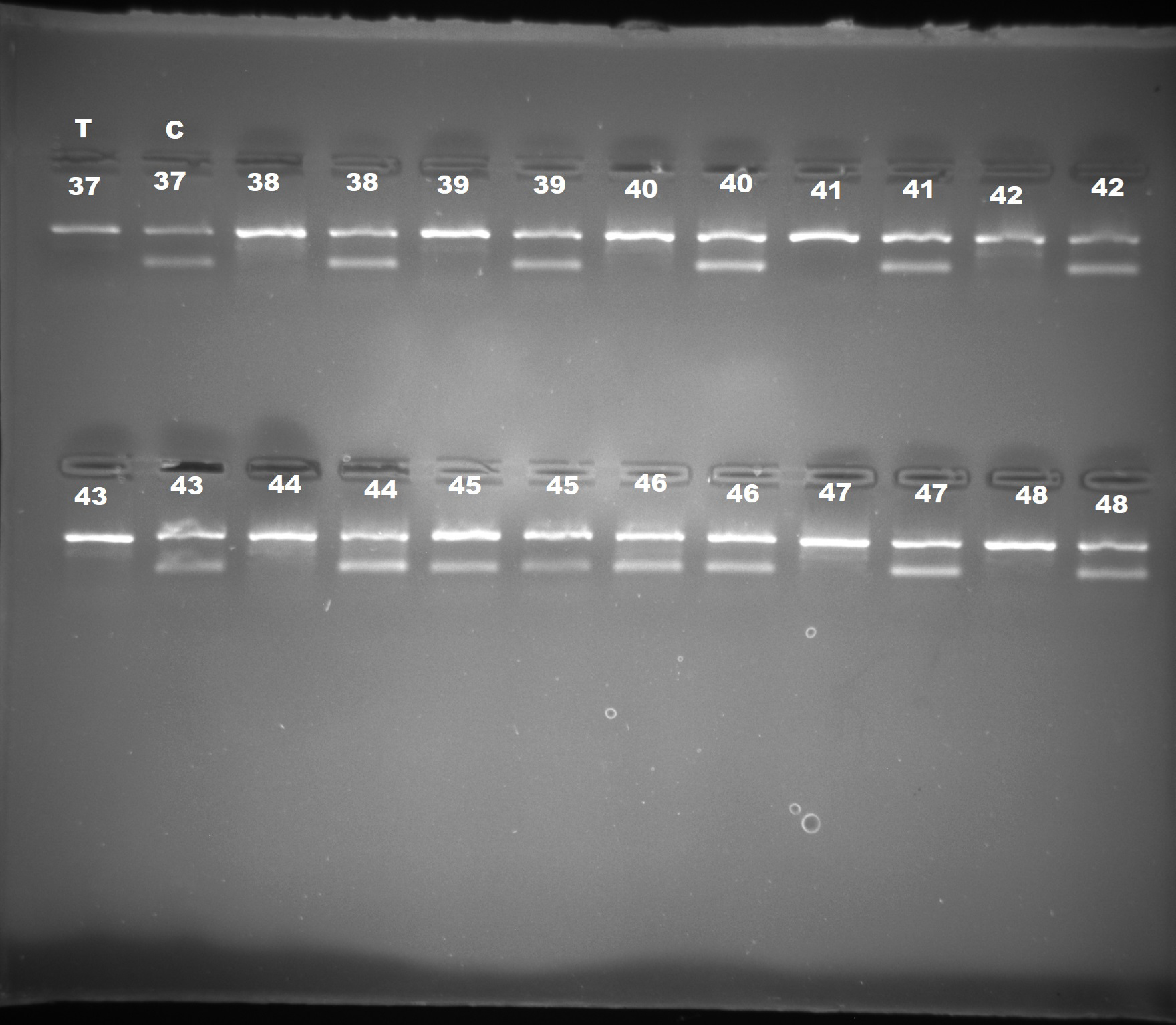
T
C
37 37 38 38 39 39 40 40 41 41 42 42
43 43 44 44 45 45 46 46 47 47 48 48

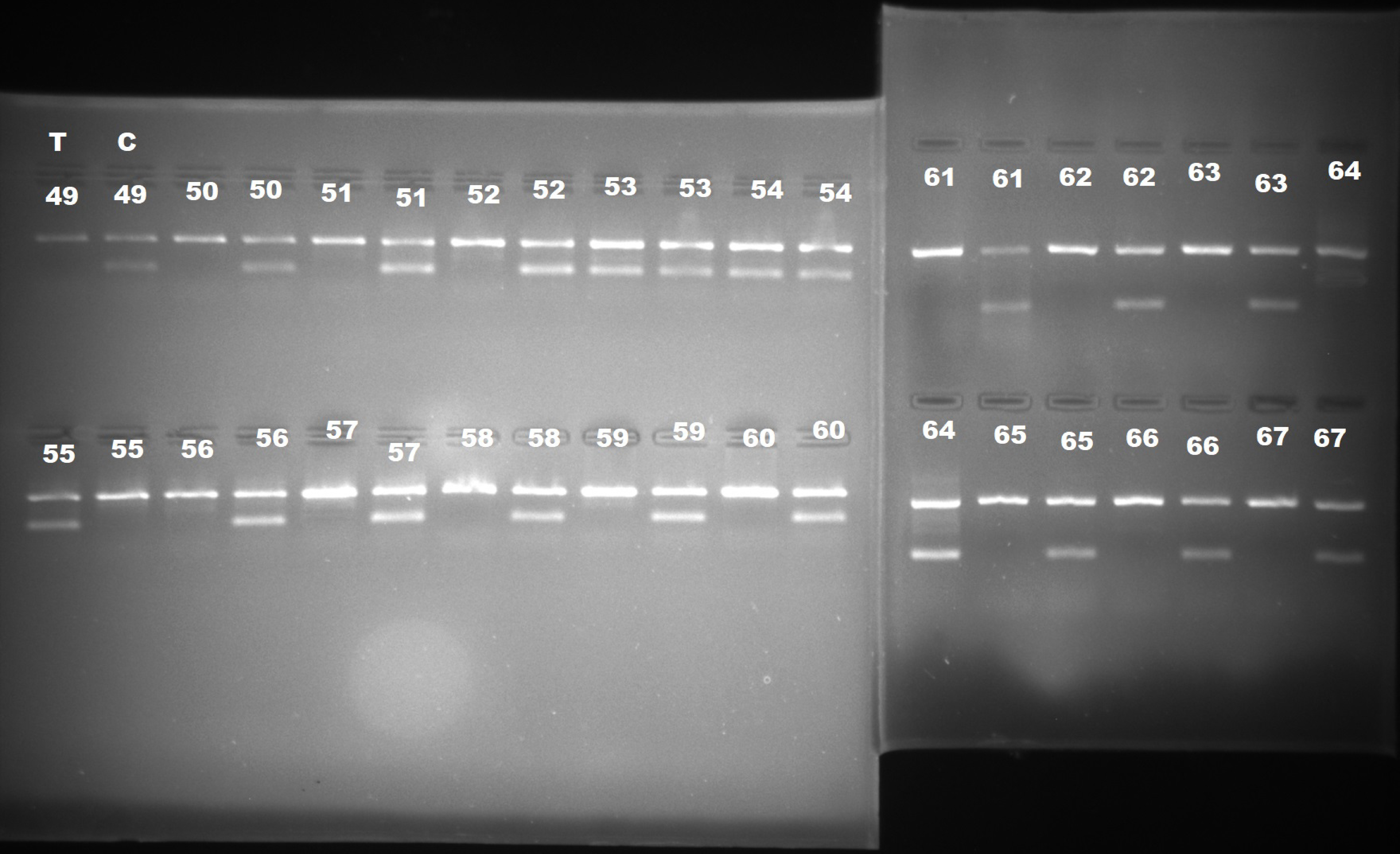
T C
49 49 50 50 51 51 52 52 53 53 54 54
55 55 56 56 57 57 58 58 59 59 60 60
61 61 62 62 63 63 64
64 65 65 66 66 67 67

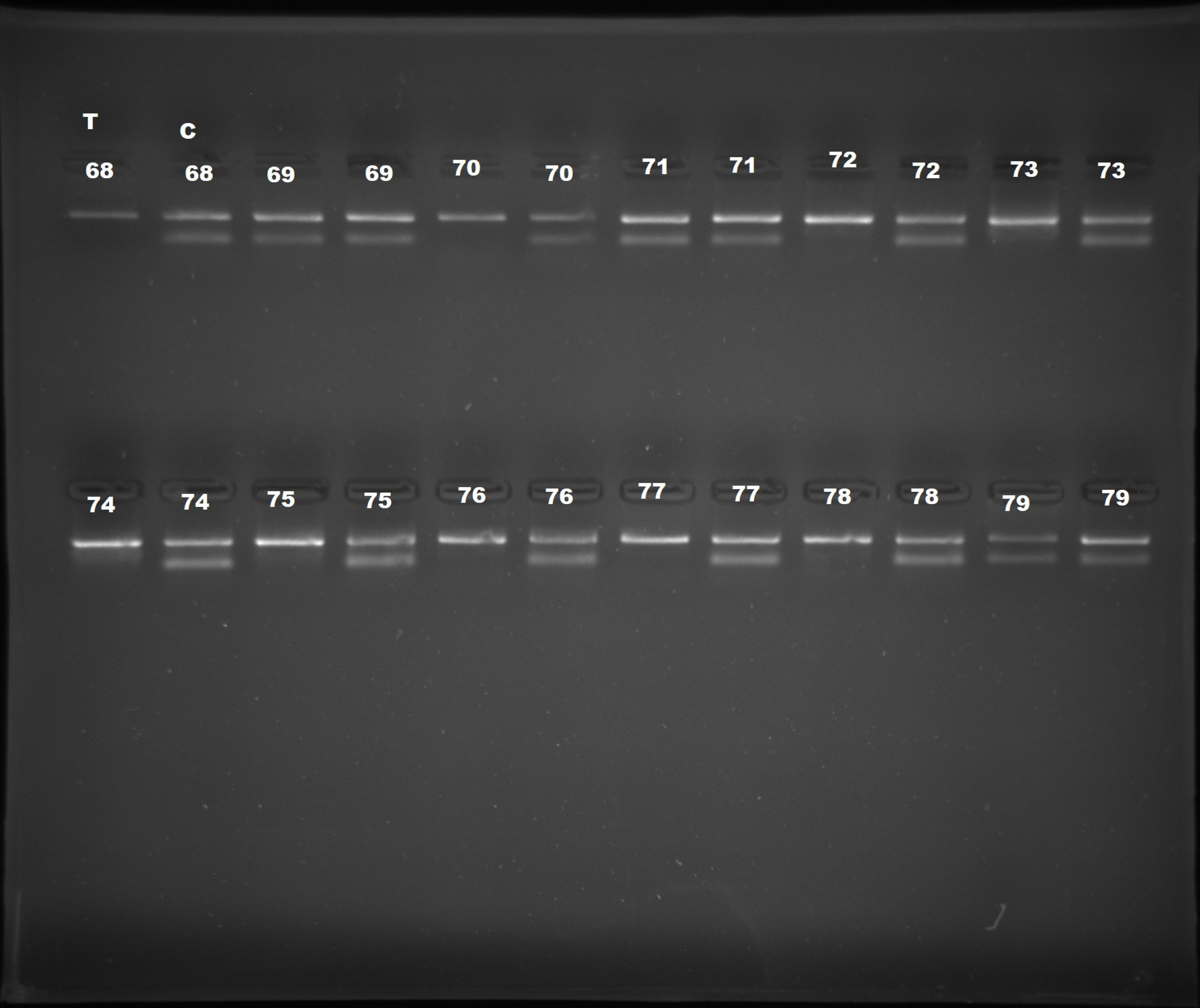
T
C
68
68
69
69
70
70
71
71
72
72
73
73
74
74
75
75
76
76
77
77
78
78
79
79

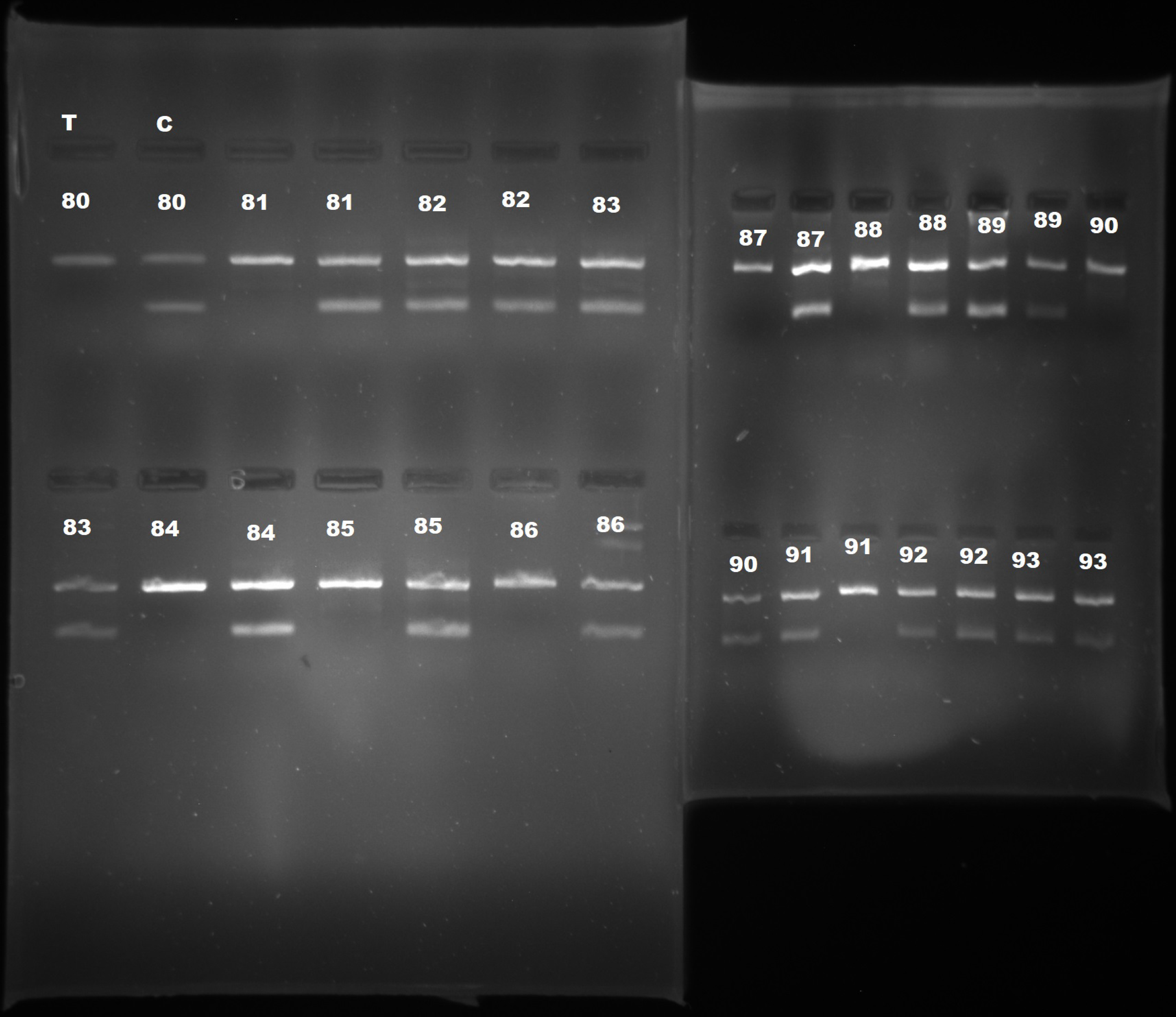
T
C
80
80
81
81
82
82
83
83
84
84
85
85
86
86
87
87
88
88
89
89
90
90
91
91
92
92
93
93

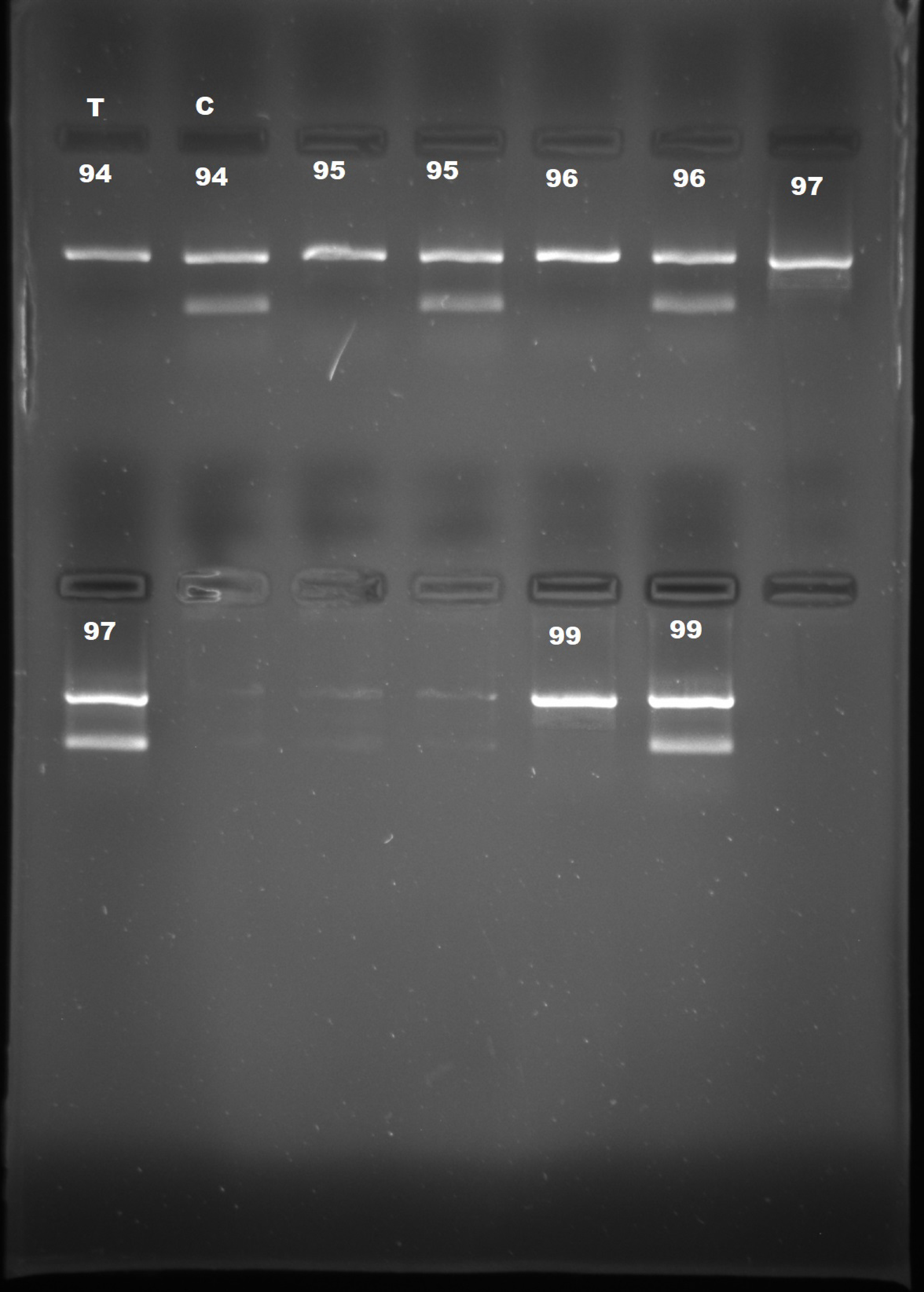
T
C
94
94
95
95
96
96
97
97
99
99

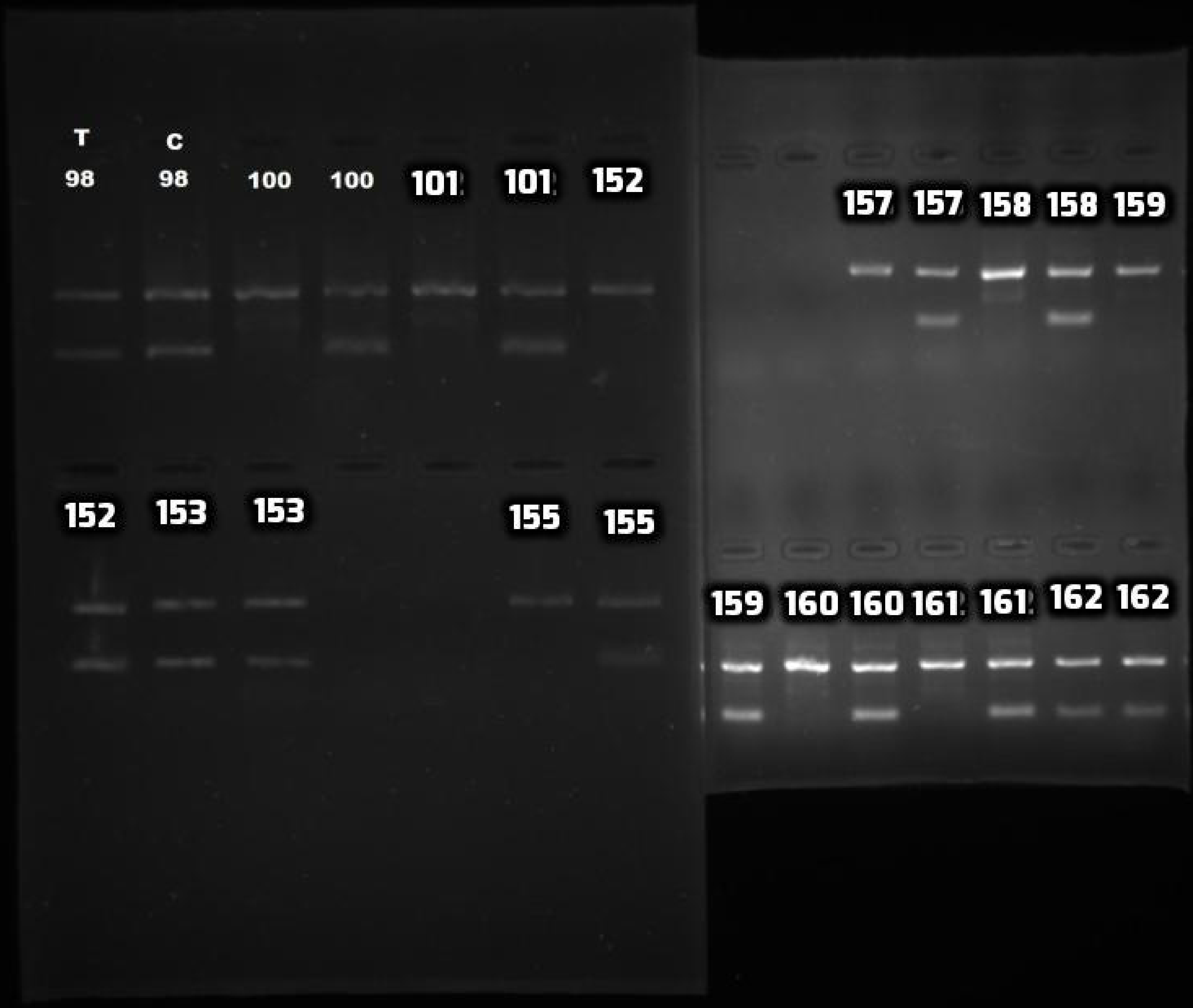
T
C
98
98
100
100
101
101
152
152
153
153
155
155
157
157
158
158
159
159
160
160
161
161
162
162

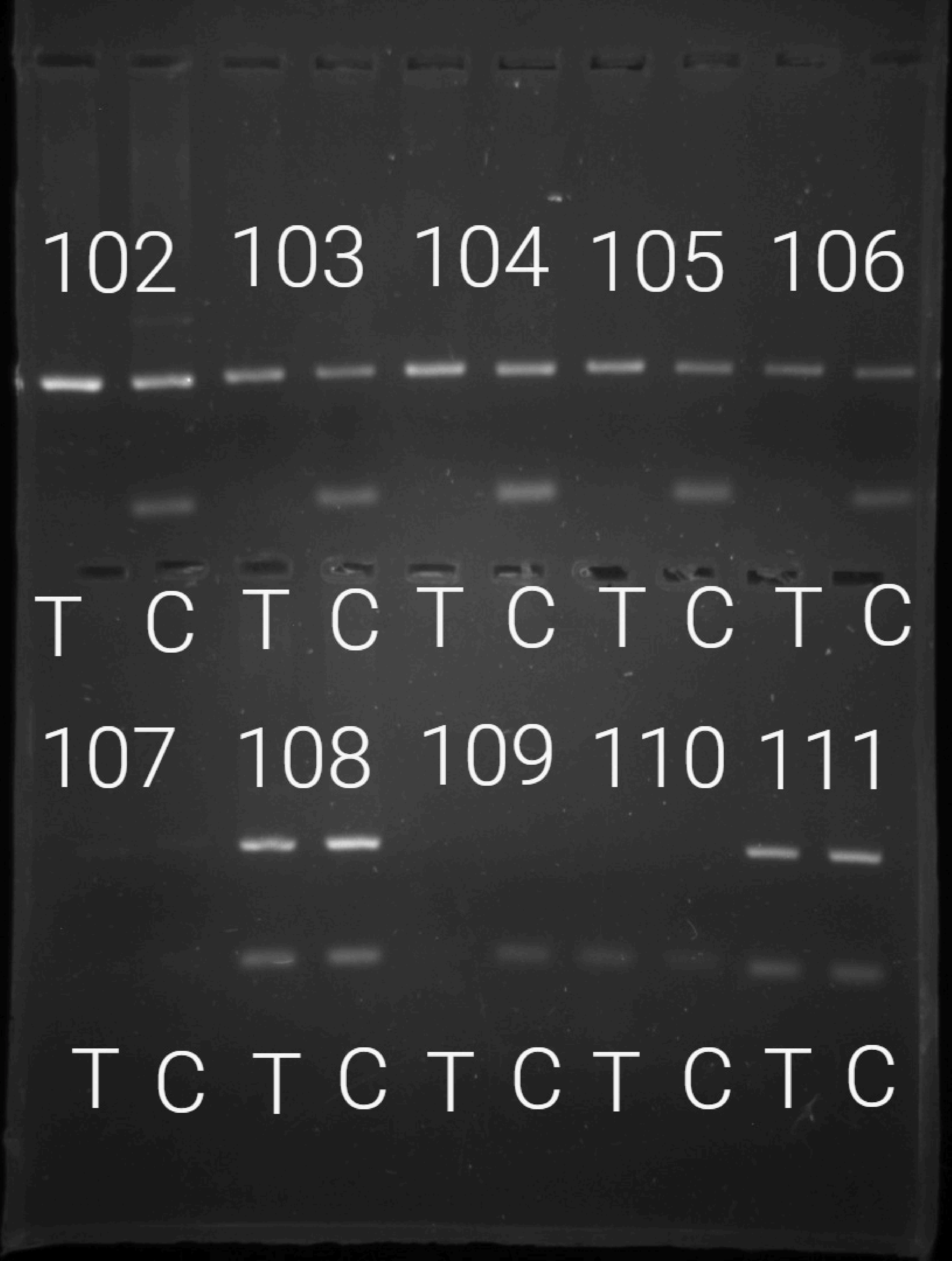
102 103 104 105 106
T C T C T C T C T C
107 108 109 110 111
T C T C T C T C T C

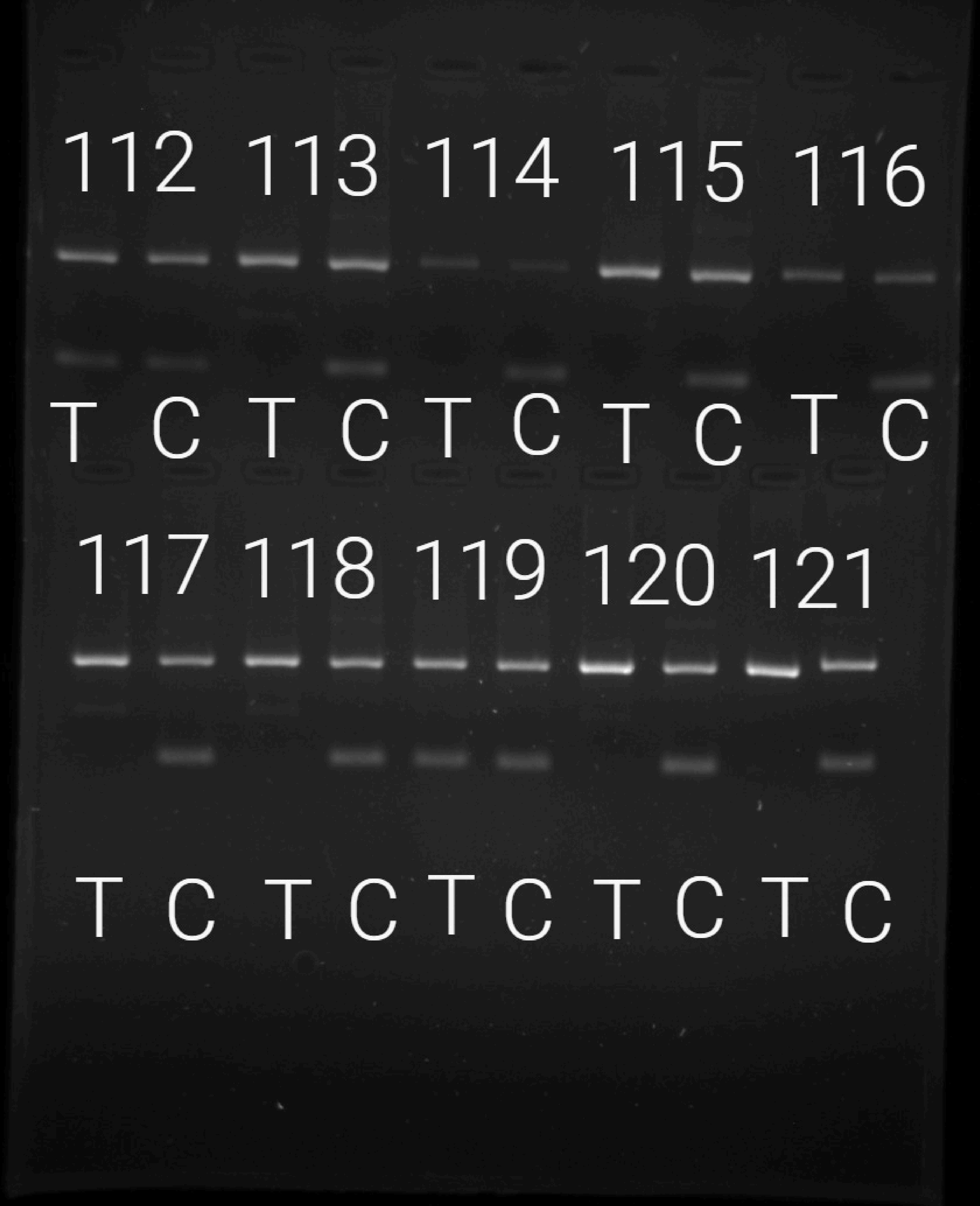
112 113 114 115 116
T C T C T C T C T C
117 118 119 120 121
T C T C T C T C T C

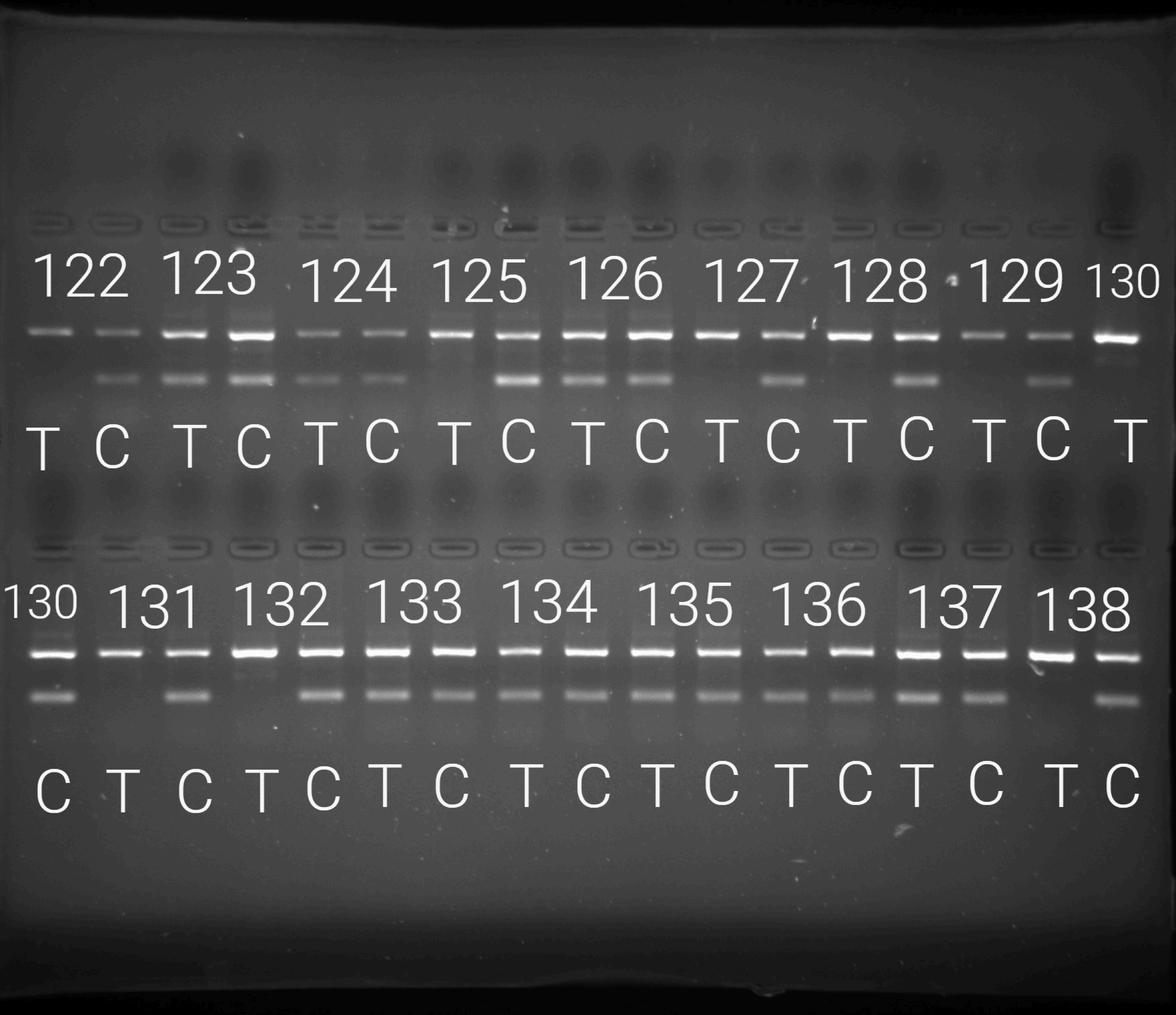
122 123 124 125 126 127 128 129 130
T C T C T C T C T C T C T C T C T
130 131 132 133 134 135 136 137 138
C T C T C T C T C T C T C T C T C

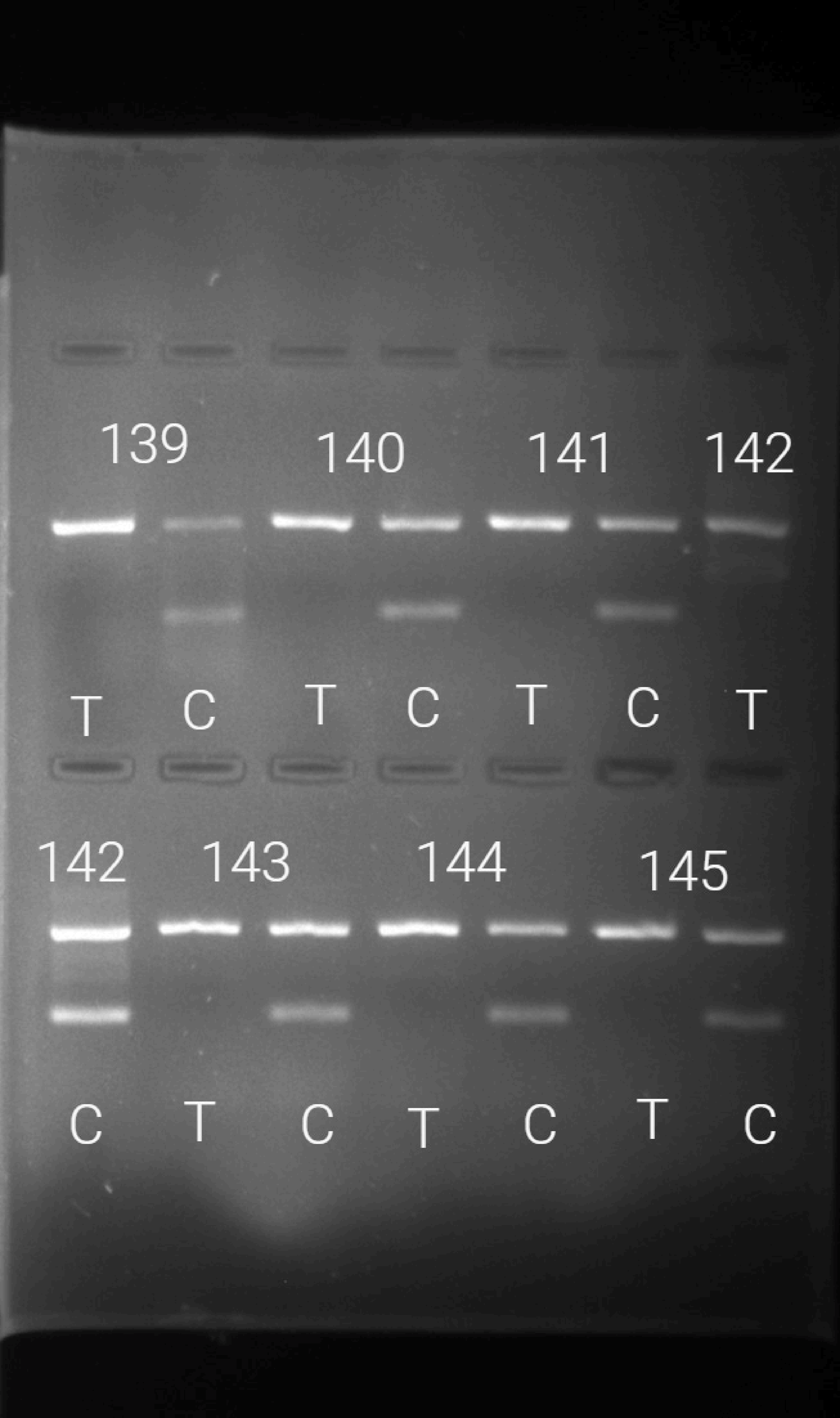
139
140
141
142
T
C
T
C
T
C
T
142
143
144
145
C
T
C
T
C
T
C

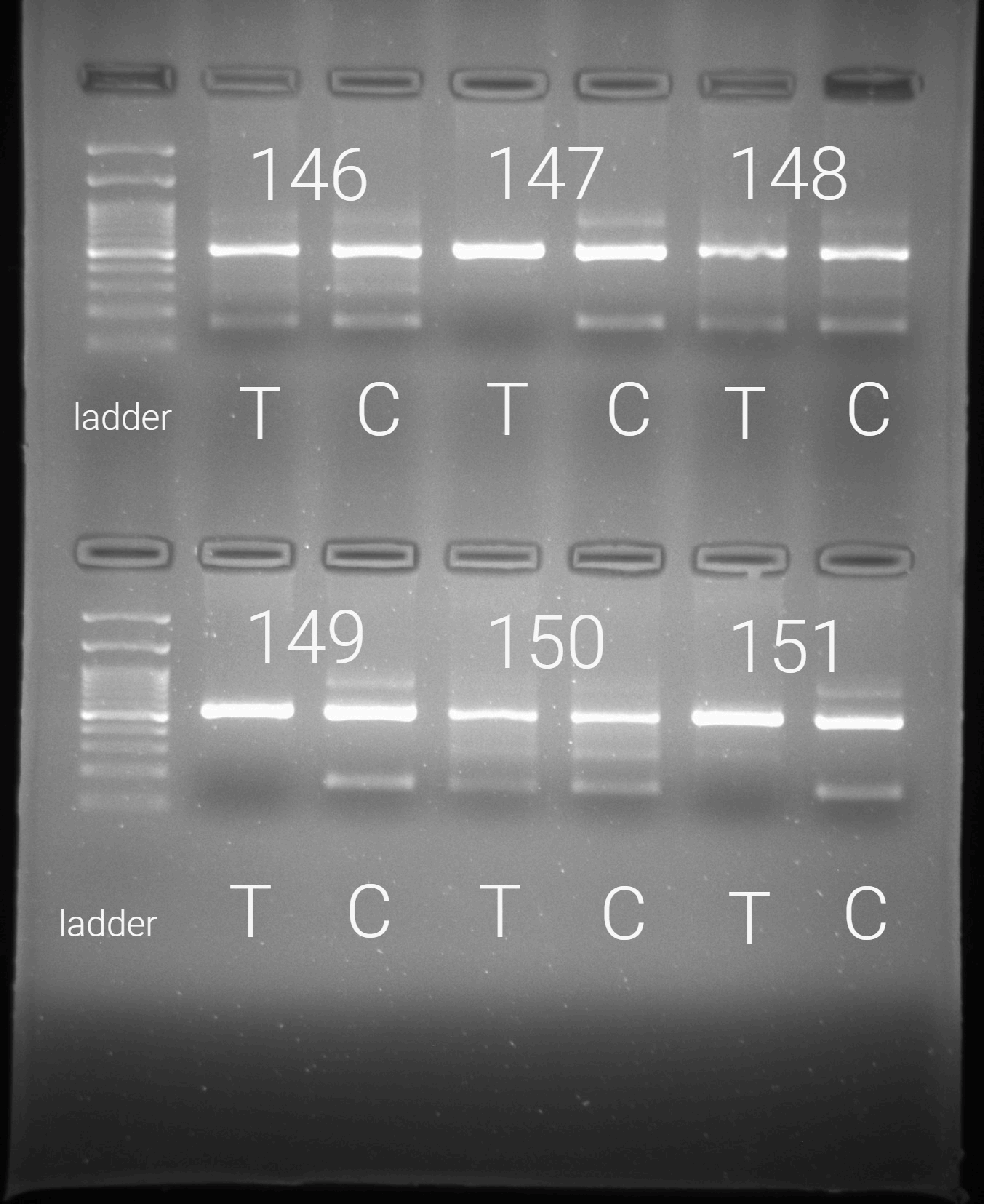
146
147
148
ladder
T
C
T
C
T
C
149
150
151
ladder
T
C
T
C
T
C

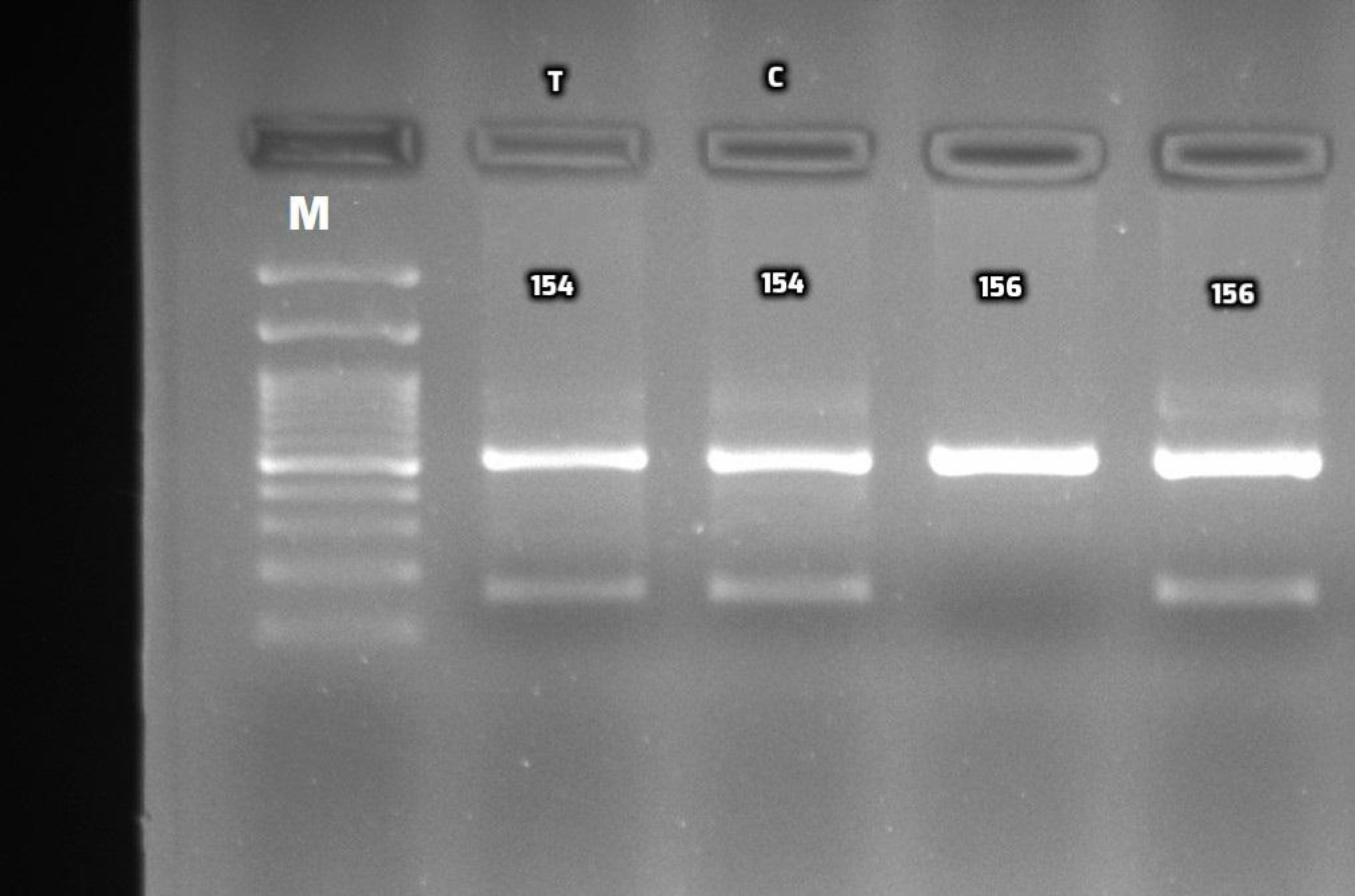
T
C
M
154
154
156
156

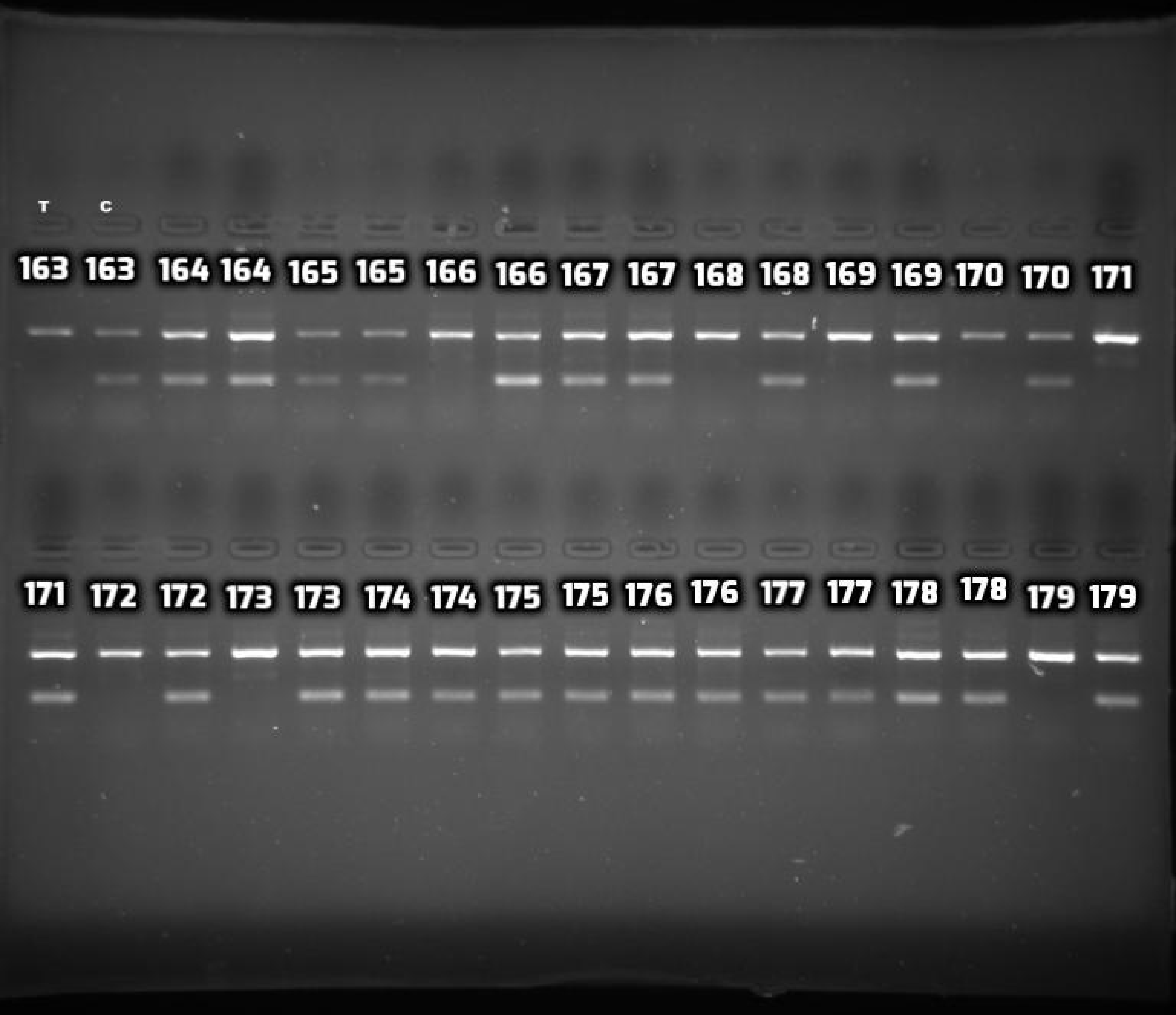
T C
163 163 164 164 165 165 166 166 167 167 168 168 169 169 170 170 171
171 172 172 173 173 174 174 175 175 176 176 177 177 178 178 179 179

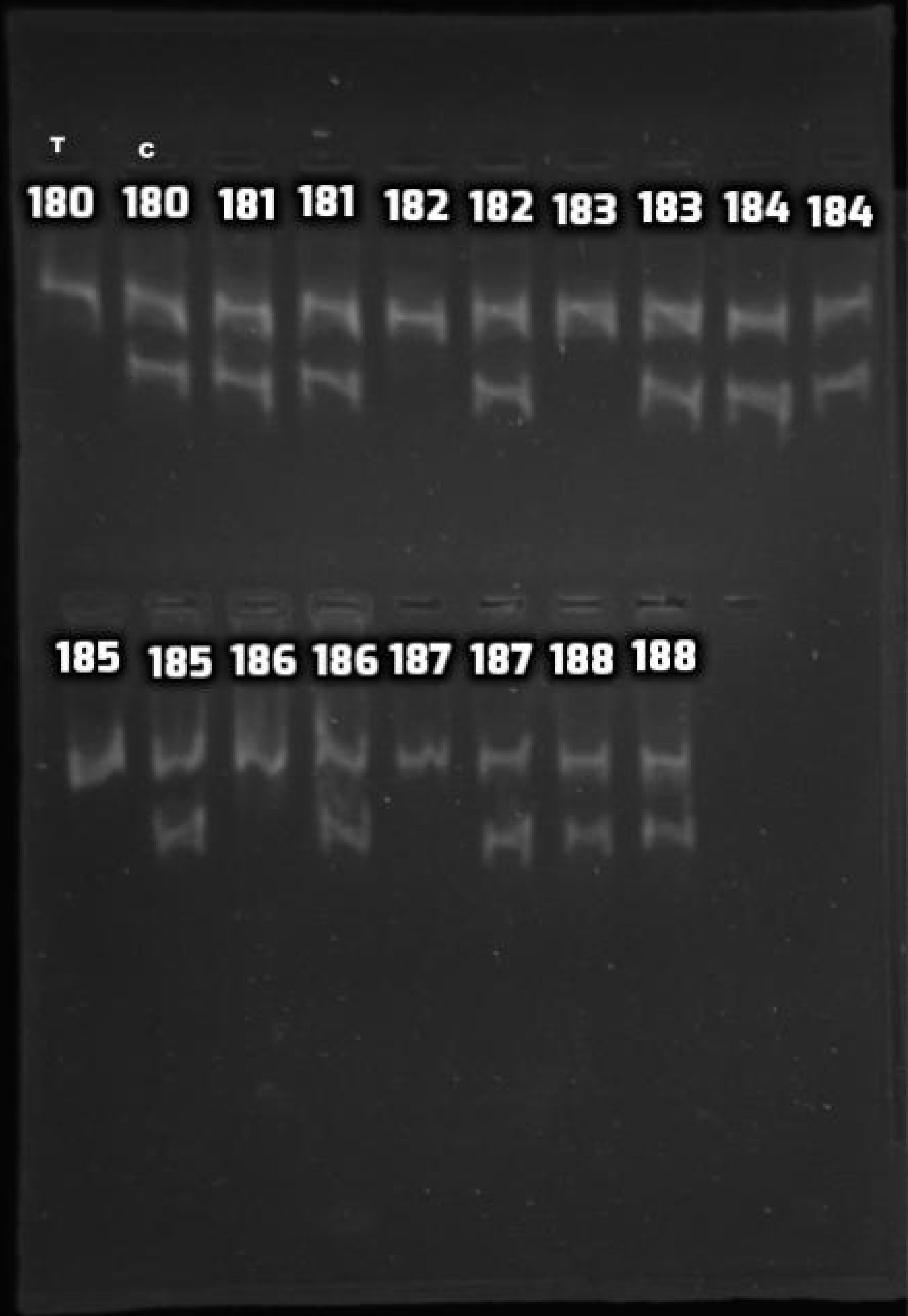
T
C
180
180
181
181
182
182
183
183
184
184
185
185
186
186
187
187
188
188

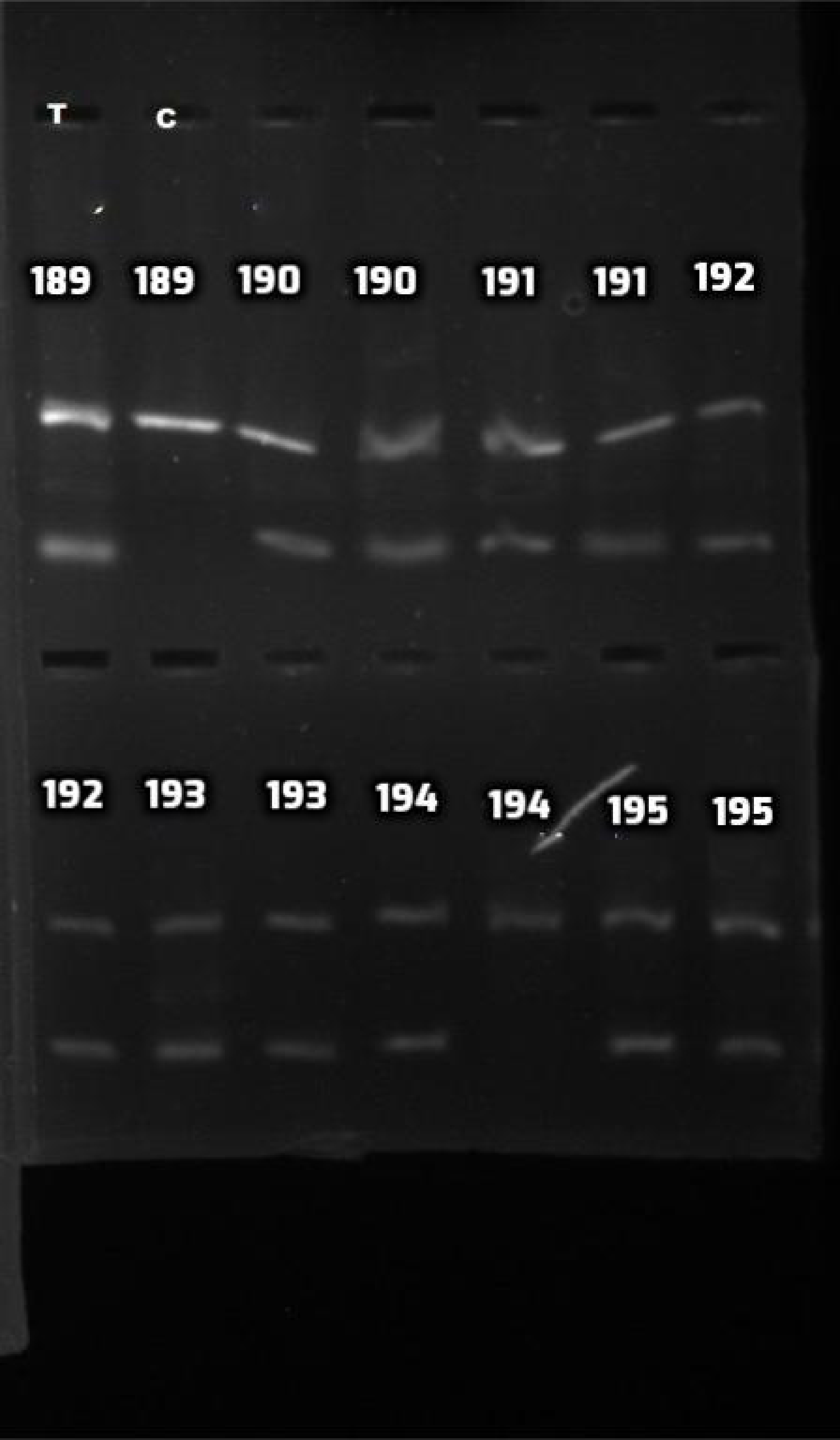
T
C
189
189
190
190
191
191
192
192
193
193
194
194
195
195

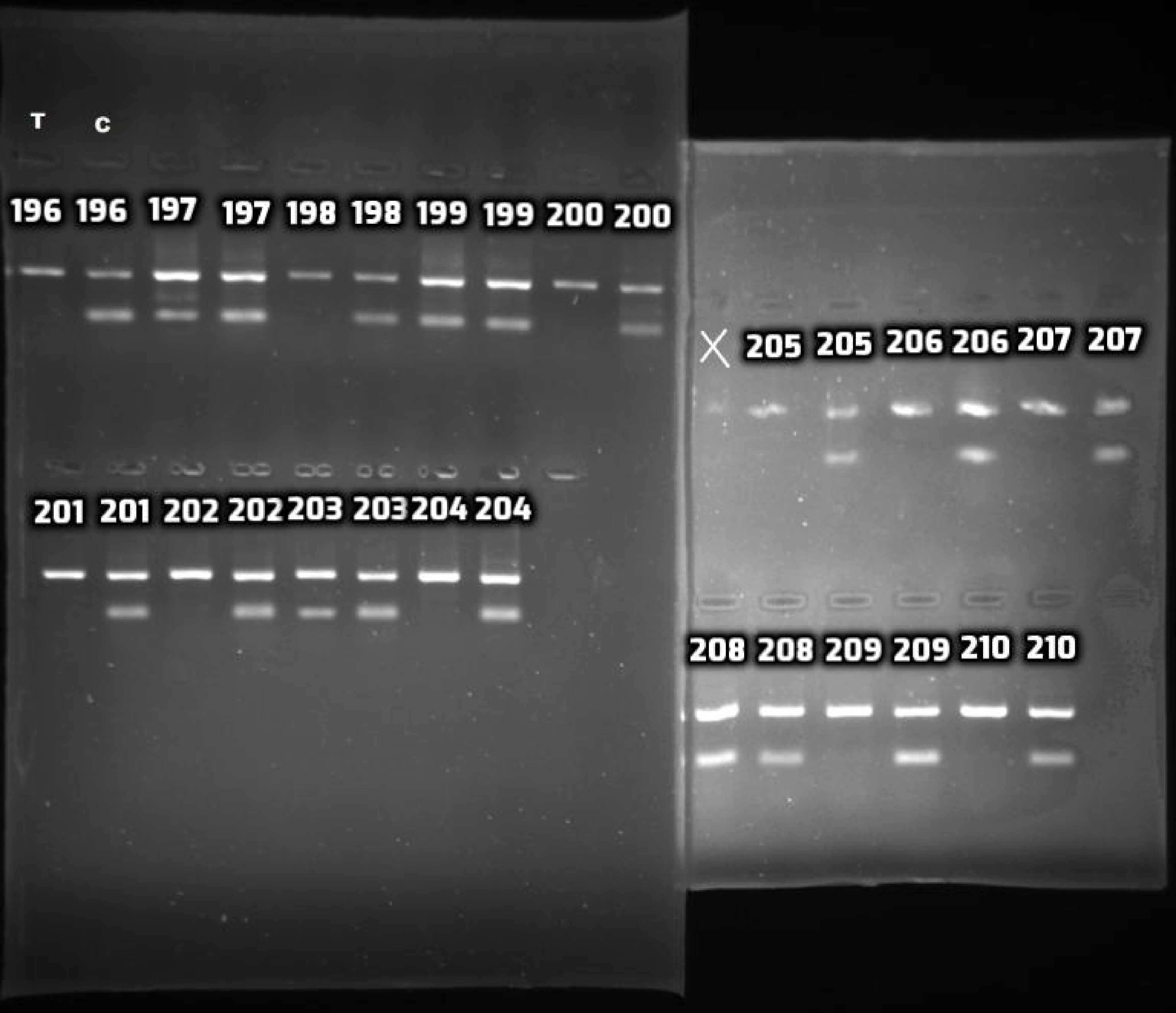
T
C
196 196 197 197 198 198 199 199 200 200
201 201 202 202 203 203 204 204
X 205 205 206 206 207 207
208 208 209 209 210 210

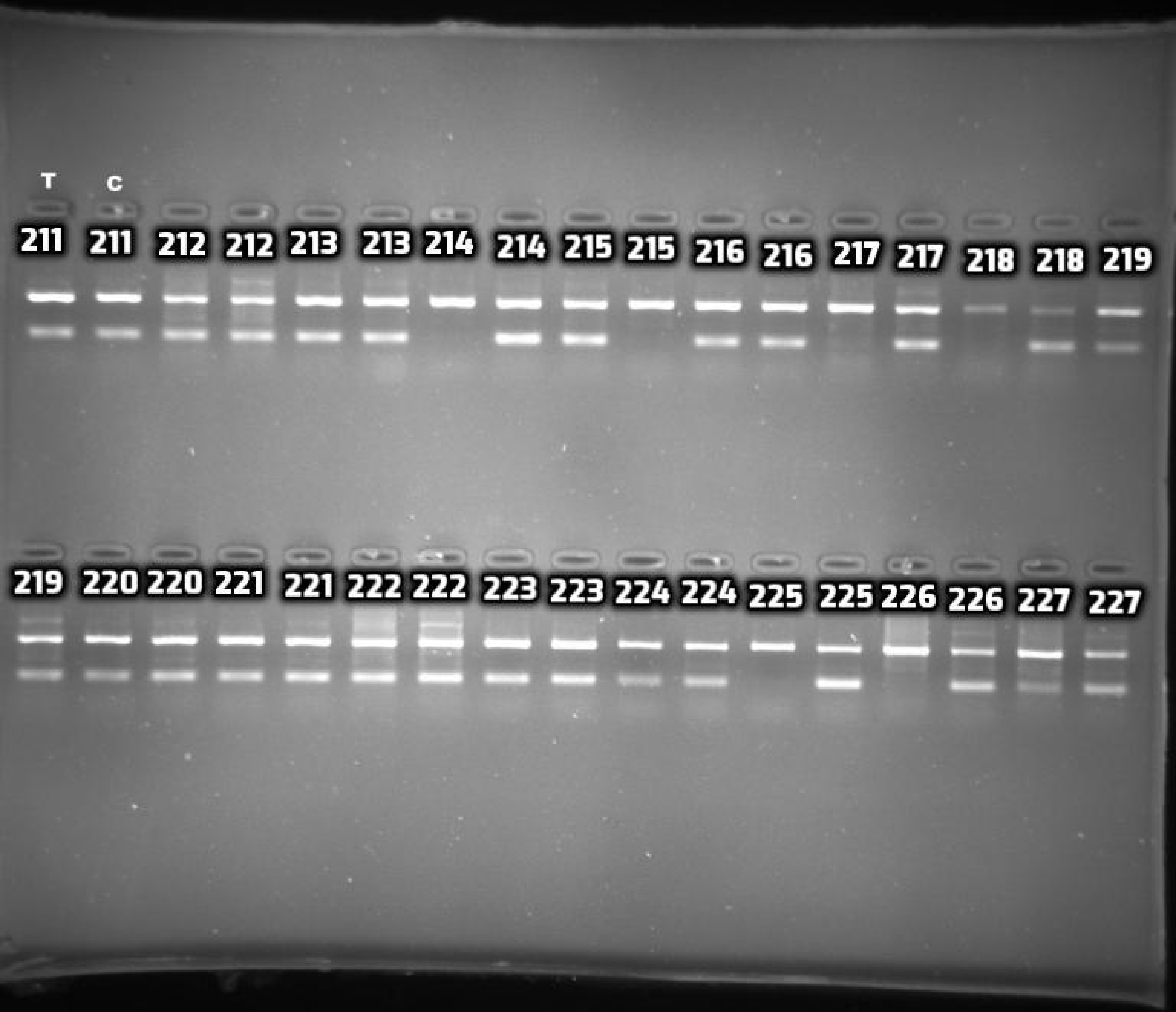
T
C
211 211 212 212 213 213 214 214 215 215 216 216 217 217 218 218 219
219 220 220 221 221 222 222 223 223 224 224 225 225 226 226 227 227

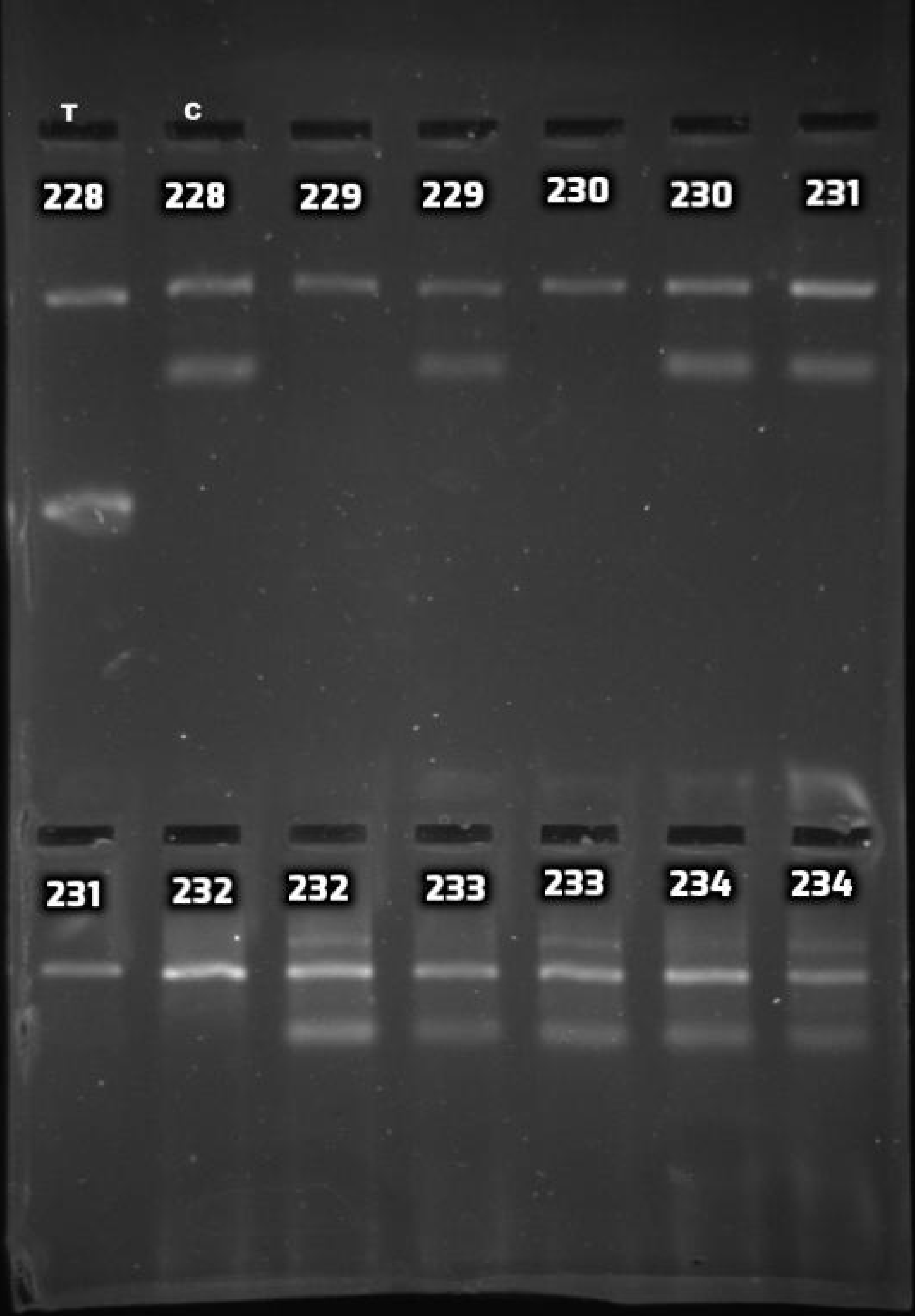
T
C
228
228
229
229
230
230
231
231
232
232
233
233
234
234

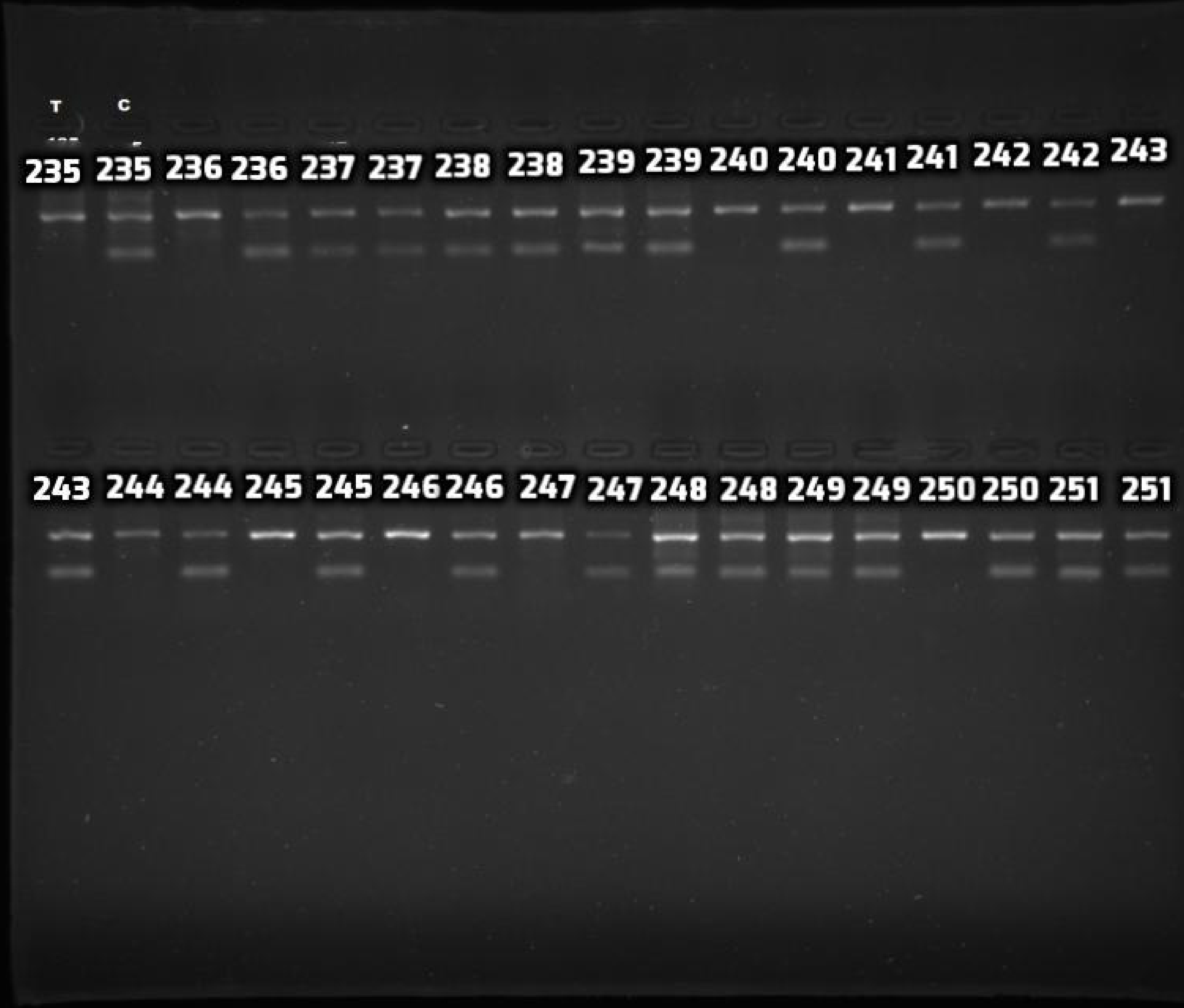
T C
235 235 236 236 237 237 238 238 239 239 240 240 241 241 242 242 243
243 244 244 245 245 246 246 247 247 248 248 249 249 250 250 251 251
